# *S100a9* might act as a modulator of the Toll-like receptor 4 transduction pathway in chronic rhinosinusitis with nasal polyps

**DOI:** 10.1038/s41598-024-60205-4

**Published:** 2024-04-27

**Authors:** Nasibeh Khayer, Maryam Jalessi, Mohammad Farhadi, Zahra Azad

**Affiliations:** 1 Skull Base Research Center, The Five Senses Health Institute, School of Medicine, Iran University of Medical Sciences, Tehran, Iran; 2 ENT and Head and Neck Research Center and Department, The Five Senses Health Institute, Rasoul Akram Hospital, School of Medicine, Iran University of Medical Sciences, Tehran, Iran

**Keywords:** Computational biology and bioinformatics, Molecular biology, Systems biology, Biomarkers, Diseases, Molecular medicine

## Abstract

Chronic rhinosinusitis with nasal polyp (CRSwNP) is a highly prevalent disorder characterized by persistent nasal and sinus mucosa inflammation. Despite significant morbidity and decreased quality of life, there are limited effective treatment options for such a disease. Therefore, identifying causal genes and dysregulated pathways paves the way for novel therapeutic interventions. In the current study, a three-way interaction approach was used to detect dynamic co-expression interactions involved in CRSwNP. In this approach, the internal evolution of the co-expression relation between a pair of genes (X, Y) was captured under a change in the expression profile of a third gene (Z), named the switch gene. Subsequently, the biological relevancy of the statistically significant triplets was confirmed using both gene set enrichment analysis and gene regulatory network reconstruction. Finally, the importance of identified switch genes was confirmed using a random forest model. The results suggested four dysregulated pathways in CRSwNP, including “positive regulation of intracellular signal transduction”, “arachidonic acid metabolic process”, “spermatogenesis” and “negative regulation of cellular protein metabolic process”. Additionally, the *S100a9* as a switch gene together with the gene pair {*Cd14*, *Tpd52l1*} form a biologically relevant triplet. More specifically, we suggested that *S100a9* might act as a potential upstream modulator in toll-like receptor 4 transduction pathway in the major CRSwNP pathologies.

## Introduction

Chronic rhinosinusitis (CRS) is a highly prevalent disorder that affects approximately 30% of adults. It is characterized by persistent inflammation of the nasal and sinus mucosa^1,2^. CRS is a heterogeneous disease that can be classified into two major subgroups with distinct pathophysiologies: CRS with nasal polyps (CRSwNP) and CRS without nasal polyps (CRSsNP)^1^. It is worth noting that up to 60% of individuals with CRSwNP also experience concurrent lower airway diseases, such as asthma, which typically onset in adulthood^3^. The main therapeutic options for CRSwNP are currently limited to intranasal glucocorticoid administration, sinus surgery, or a combination of both^4^.

Despite the significant morbidity and negative impact on quality of life, our understanding of the underlying molecular mechanisms and specific biomarkers associated with CRSwNP remains incomplete^5^. Therefore, identifying causal genes and dysregulated pathways paves the way for novel therapeutic interventions.

Advances in high-throughput gene expression profiling technologies, such as microarray and RNA sequencing, have provided unprecedented opportunities to produce disease-related transcriptome datasets^6^. Such datasets provide a genome-scale snapshot of gene expression profiles, serving as invaluable resources for deducing gene interactions. Moreover, the identification of disease-related gene interaction plays a pivotal role in unraveling the intricate molecular mechanisms as well as identifying the critical genes in a specific disease. Furthermore, the conclusions drawn from the same biological data can vary based on the computational approach employed^7,8^.

In a few previous studies, transcriptome datasets have been investigated to identify the pathogenicity mechanisms and potential drug targets in CRSwNP. Recently, Peng et al. performed a comprehensive genome-wide gene expression analysis in order to elucidate the pathways and candidate gene sets associated with CRSwNP. They investigated the differentially expressed genes and corresponding pathways in a CRSwNP-related dataset. Their results suggested several biological pathways involved in CRSwNP, including “defective host defences”, “inflammation” and “abnormal metabolism of extracellular matrix”^9^. In another recent study, Hao et al. employed an integrated analysis approach to investigate the dysregulated pathway as well as the crucial genes in CRSwNP from a diagnostic and therapeutic perspective. Their results indicated several CRSwNP-related pathways, including “immune effector process”, “leukocyte migration”, “regulation of the inflammatory response”, “*Staphylococcus aureus* infection”, and “cytokine-cytokine receptor interaction”. Moreover, they suggested *Alox5ap*, *Bcl2a1*, *Btk*, *Cybb*, *Ncf2*, *Hck*, and *Hk3* genes, which significantly increase in CRSwNP patients, as potentially crucial genes in the pathogenesis^10^.

A predominant feature of gene interactions is often highly dynamic, invariably linked to alterations in cellular conditions as a reaction to diverse external stimuli and signals^11^. In other words, a pair of genes participating in a comparable biological pathway during a particular biological condition might lose their connection under a different biological circumstance. As a result, the degree and pattern of gene expression correlation between a pair of genes can be influenced by internal changes and cellular conditions^12^. Therefore, pairwise interaction analyses, in the classical sense, may be too simplistic to explain complex molecular relationships^13,14^.

The three-way interaction approach describes the internal evolution of the co-expression relation between a pair of genes (X, Y)^13,15^. If it is supposed that a state change within a cell is associated with the expression levels of a third gene, say Z, the expression profile of Z can capture the dynamic nature of the co-expression relation of two genes X and Y. Indeed, the Z gene as a surrogate for the intrinsic-state variable control the evolution of the co-expression relation of X and Y genes. Therefore, such an approach possesses the capability to decipher sophisticated molecular relations at an elevated level of complexity compared to the classical pairwise interaction approach^16,17^. From now on, such triplet will be shown as Z/{X, Y}.

So far, the three-way interaction approach has been employed to deciphering the pathogenesis of several diseases, including inflammatory diseases^18,19^. The importance of *RT-DOb*/{*Csf1r*, *Milr1*} triplet in onset of Alzheimer’s disease has been suggested and validated in previous studies. These studies indicated the change in expression level of *RT-DOb* gene, as a switch gene, can disturb the communication between mast cell and microglia^19,20^. Another study suggested the *Rps27a* gene can act as a switch gene for {*Il-18*, *Cx3cl1*} gene pair and be associated with pathogenesis of the major neurodegenerative diseases by controlling the microglia activation^21^.

The objective of the present study is a comprehensive analysis of CRSwNP-related transcriptome data, aiming to shed light on dysregulated molecular mechanisms as well as to trace the critical therapeutic targets in the CRSwNP. For such purpose, we used a novel computational method, i.e., liquid association (LA), as a powerful tool to capture dynamic co-expression relationships^15^.

## Results

### Statistically significant three-way interaction determination

The liquid association measure was computed for every possible Z/{X, Y} combination in the dataset. The top 200,000 triplet combinations with the highest significance based on *p*-value were selected as primary outputs.

**Figure 1:**
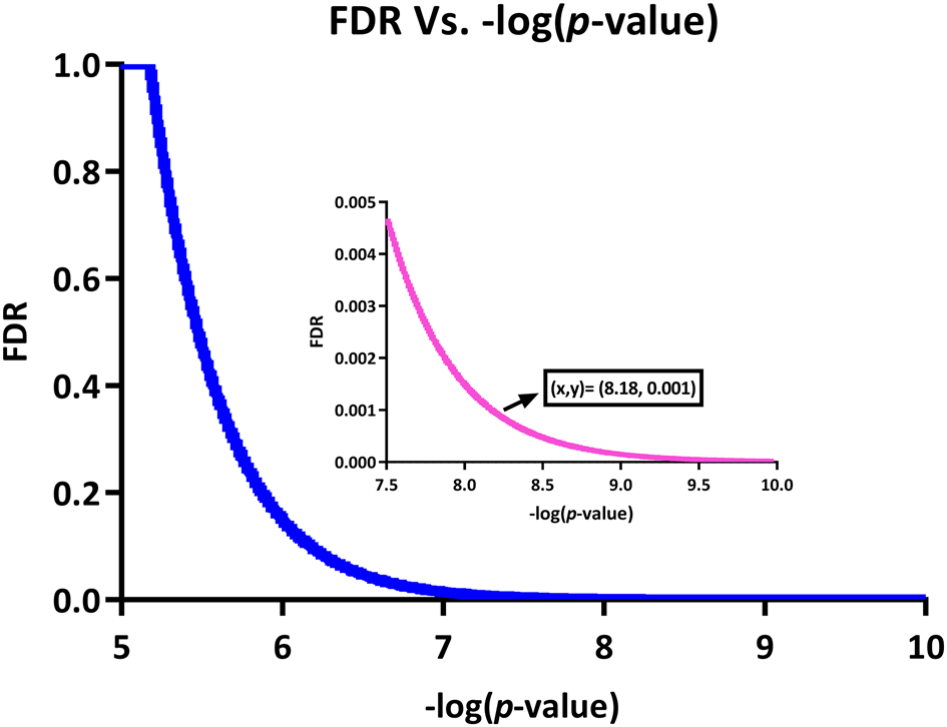
FDR vs. − log (*p*-value). The changes in FDR (BH-corrected p-value) versus − log (*p*-value) for the first 200,000 results of fastLA. As shown, FDR = 0.001 corresponds to − log (*p*-value) = 8.18.

A *p*-value histogram of such triplet combinations is presented in Supplementary Fig. S1 online. Moreover, Fig. 1 demonstrates a downward trend of adjusted *p*-values versus − log (*p*-value).

**Figure 2:**
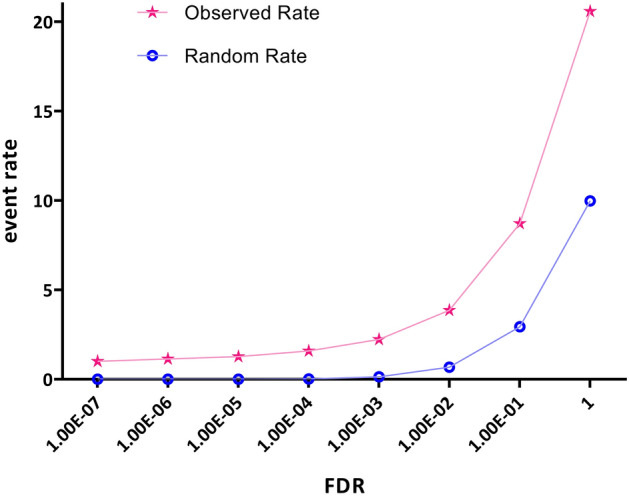
Evaluation of the fastLA analysis accuracy. This figure presents an assessment of the accuracy of the fastLA analysis. The study investigates the relationship between significant fastLA *p*-values and the observed event rate of genes located at position Z (switch) in comparison to the random event rate. The findings unequivocally show a substantial deviation between the observed event rate of switch genes and the random rate, providing strong evidence to support the accuracy of the fastLA analysis.

In order to assess the accuracy of liquid association analysis, the observed event rate of Z position (switch) genes was compared with the random event rate across a broad range of significant fastLA *p*-values. Figure 2 presents this comparison, which contributes to assessing the analysis's reliability.

A subset of triplet combinations was chosen for further analysis based on an adjusted *p*-value threshold of less than 10^−3^ (Fig. 1A). This subset consisted of 807 statistically significant triples. An explanation about the chosen cut-off point in this analysis has been presented in the Supplementary Text S1 online. Moreover, the complete list of all statistically significant triplets can be found in the Supplementary Table S1 online.

### Biologically relevant three-way interaction identification

GSEA was conducted as the first step to identify biologically relevant triplets. This analysis focused on the 807 statistically significant triplets. Because of the generality of terms in lower levels of gene ontology, only terms in levels higher than 6 were included. The complete list of enriched terms by considering BH-corrected *p*-value < 0.05 is available in the Supplementary Table S2 online.

Consistent with the three-way interaction model concept, it is expected that in a biologically-relevant triplet, the X and Y genes would be involved in the same biological process. In light of this, the analysis focused on identifying statistically significant triplets and examining their association with enriched terms to ascertain their biological relevance.

As the next step in uncovering biologically relevant triplets, the reconstruction of the GRN was performed using the ARACNE algorithm. Then, the regulatory relationships involving statistically significant triplets were investigated while disregarding any relationships with more than three mediators.

**Figure 3:**
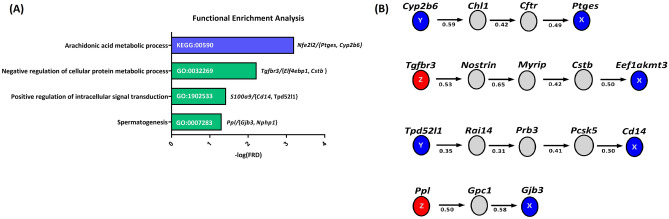
The biological relevance of four statistically significant triplets. (**A**) The enriched biological process and pathway terms corresponding with four biologically relevant triplets have been presented in this figure. (**B**) This figure illustrates the positioning of four biologically relevant triplets within the Gene Regulatory Network (GRN). The red nodes represent the genes located at the Z position within each triplet, while the green nodes represent the genes located at the X and Y positions. Additionally, other genes are depicted as grey nodes.

Collectively, using both GSEA and GRN analyses, the biological relevance of four statistically significant triplets was verified. These triplets consist of *S100a9*/{*Cd14*, *Tpd52l1*} triplet, *Nfe2l2*/{*Ptges*, *Cyp2b6*} triplet, *Ppl*/{*Gjb3*, *Nphp1*} triplet, and *Tgfbr3*, {*Elf4Ebp1*, *Cstb*} triplet, which are involved in the biological processes of “positive regulation of intracellular signal transduction”, “arachidonic acid metabolic process”, “spermatogenesis” and “negative regulation of cellular protein metabolic process”, respectively (Fig. 3).

**Figure 4:**
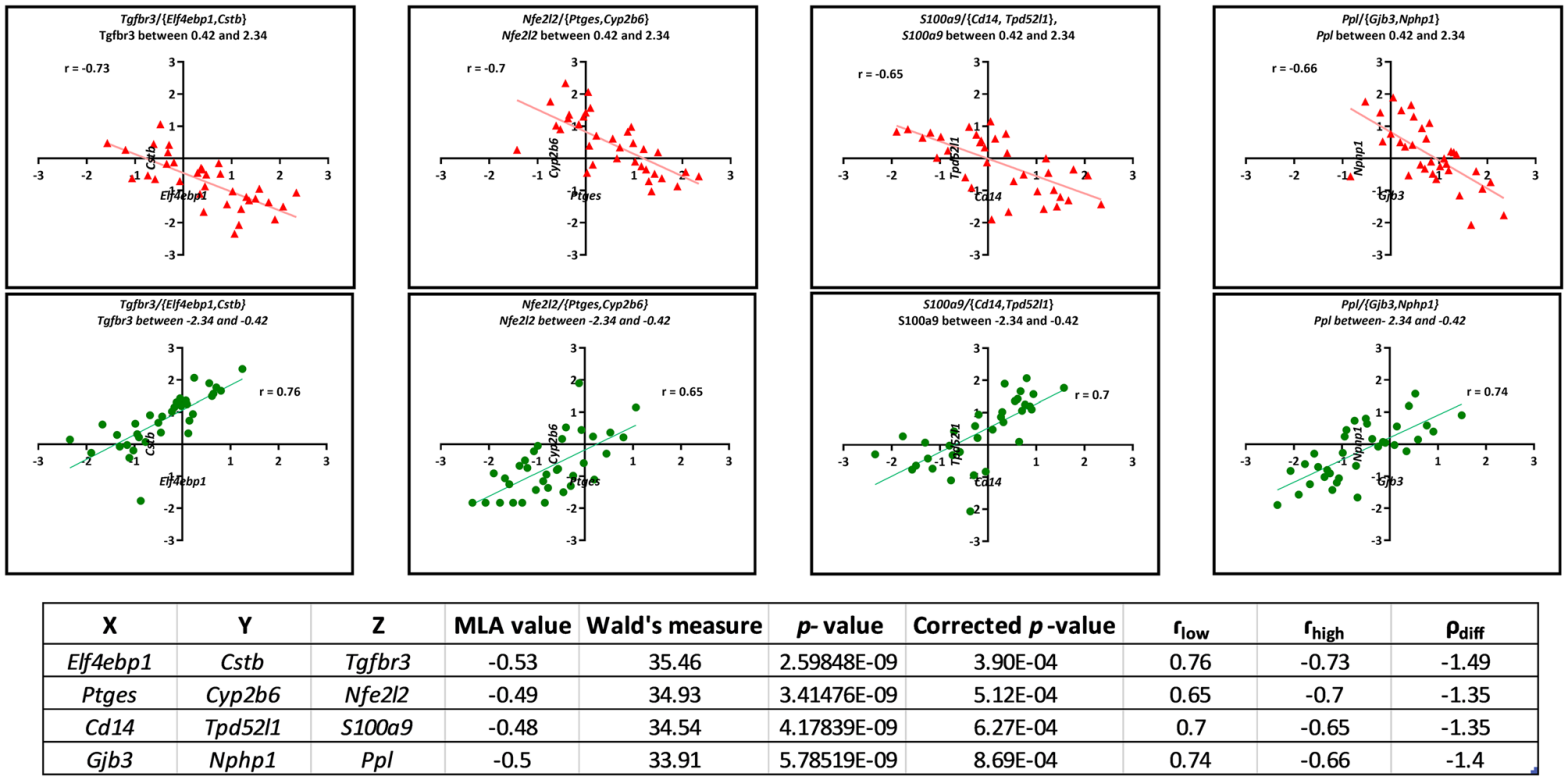
Scatter plots of four triplets that are biologically relevant. There is a notable change in the correlation between X and Y due to a change in the expression level of Z. The observed alterations emphasize the significant influence of Z expression on the relationship between X and Y, highlighting its biological relevance within the context of the studied system.

Moreover, the scatter plots of these triplets are presented in Fig. 4, depicting a notable correlation change between X and Y in response to variations in Z expression levels within three different ranges.

### Random forest and selecting important genes

**Figure 5:**
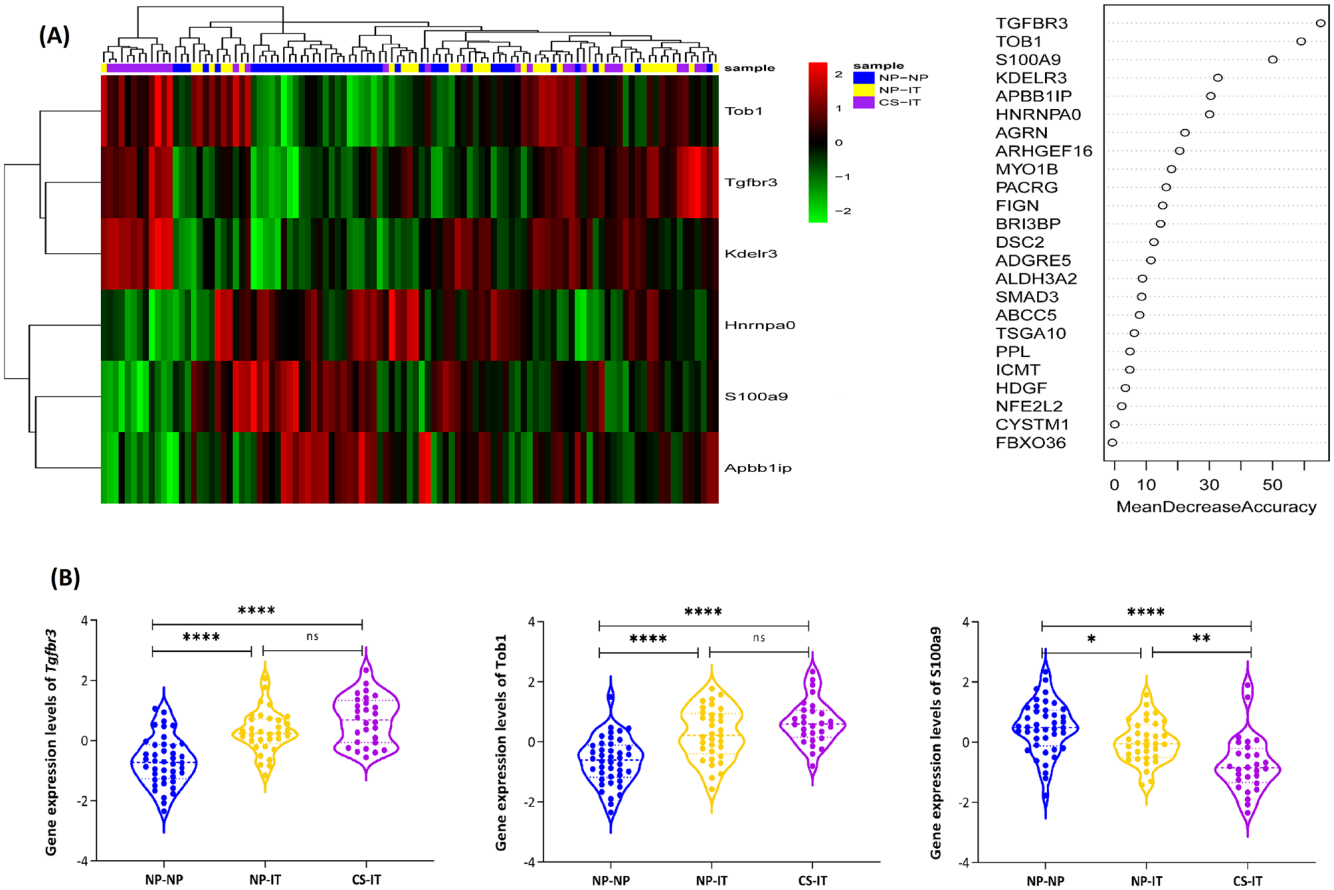
(**A**) Random forest classification and selection of important genes. The genes were chosen based on the Mean Decrease Accuracy measure, which serves as a reliable metric for determining their significance in the classification model. The 6 top genes that exhibit the highest importance in contributing to the accuracy of the random forest classification have been presented. (**B**) Results of Tukey’s Honest Significant Difference (HSD) analysis. This bar graph illustrates the outcomes of the Tukey's HSD test on the gene expression levels of *Tgfbr3*, *Tob1*, and *S100a9* across three experimental groups: NP-NP, CS-IT, and NP-IT. Asterisks above the error bars indicate significant differences between group means as determined by Tukey’s HSD test, with significance levels marked as follows: *p < 0.05, **p < 0.01, ***p < 0.001, ****p < 0.0001. The gene expression data for each group is represented in distinct colors to enhance visual distinction.

The random forest algorithm offers various metrics for assessing variable importance, among which Mean Decrease Accuracy (MDA) is considered the most reliable. MDA determines importance by evaluating the reduction in classification accuracy when the expression values of a particular gene are randomly permuted^18,19^. In our study, we utilized MDA to identify the important switch genes, as presented in Fig. 5A. The results showed that *Tgfbr3*, *Tob1* and *S100a9* genes are more important in classifying such groups.

Furthermore, the area under the receiver operating characteristic (ROC) curve (AUC) is widely employed to evaluate the performance of supervised classification models^20^. In our analysis, we utilized ROC curves to assess the sensitivity and specificity of the Random Forest model. The results, illustrated in Supplementary Fig. S3 online, indicate an AUC of 0.87, a sensitivity of 95, and a specificity of 46 for the classifier.

The ANOVA results reveal significant differences in the gene expression profiles of three specific genes (*Tgfbr3*, *Tob1*, and *S100a9*) across various groups (Supplementary Tables S4–S6). Tukey’s HSD analysis further indicates that the expression levels of these genes significantly differ between the NP-NP group and the CS-IT group, as well as between the NP-IT group and the CS-IT group. Notably, the *S100a9* gene also demonstrates a significant difference between the NP-IT and CS-IT groups, as shown in Fig. 5B. Detailed gene expression profiles for these genes can be found in Supplementary Table S7.

### Identifying novel putative drugs

In a biologically relevant triplet, the Z gene is prone to be used as a drug target because this gene can control the intrinsic state changes associated with a disease. Hence, in order to determine novel potential drugs, four switch genes belonging to the biologically relevant triples, including *S100a9*, *nfe2l2*, *Ppl* and *Tgfbr3*, were explored in drug-related databases.

The results identified two drugs, namely “tasquinimod” and “paquinimod”, are associated with the *S100a9* genes and two corticosteroid drugs, namely “dexamethasone” and “regramostim”, are related to the *Tgfbr3* gene.

## Discussion

Chronic rhinosinusitis (CRS) is an inflammatory disease severely affecting the nasal mucosa^2^. Nasal polyps (NP) are a common comorbidity, impacting around 30% of CRS patients, leading to significant socioeconomic burdens and reduced quality of life^22^. The clinical management of CRSwNP could be more efficacious by increasing the knowledge of the underlying pathogenic factors and subsequently entering effective therapeutic interventions^4^. Therefore, the LA analysis was employed in this study to capture critical triplets associated with CRSwNP pathogenesis. To our knowledge, the CRSwNP transcriptome data has yet to be investigated through the 3WI model.

The accuracy of the LA assay was verified by comparing the observed occurrence rate of Z position genes within a wide range of significant fastLA *p*-values, juxtaposed against a randomly determined occurrence rate. It is expected that there will be a notable decrease in the number of genes occupying the Z position compared to what would be expected by chance. This expectation arises from the fact that a relatively small set of genes governs the majority of biological processes. As shown in Fig. 2, the observed event rate for switch genes is markedly far from what would be expected by chance. The results showed that specific genes predominantly occupy the Z positions within statistically significant triplets.

### The relationship between enriched pathways and CRSwNP

The pathway enrichment analysis results (Fig. 3) show that four pathways are involved in CRSwNP. These pathways include “positive regulation of intracellular signal transduction”, “arachidonic acid metabolic process”, “spermatogenesis”, and “negative regulation of cellular protein metabolic process”. In the following, the relationship between these pathways and CRSwNP is explained in detail. See below.

The “intracellular signal transduction” plays a crucial role in the regulation of cellular processes such as proliferation, differentiation, and apoptosis^23^. Dysregulation of intracellular signaling pathways has been implicated in the pathogenesis of several diseases, including chronic rhinosinusitis with nasal polyps (CRSwNP)^24–26^. Several studies have suggested the role of intracellular signal transduction pathway in the development and progression of CRSwNP. Spleen tyrosine kinase (Syk) plays a significant role in intracellular signal transduction in various types of hematopoietic cells^27^. According to a study, the expression of the Syk gene is increased in patients with allergic nasal polyps^28^. Another study suggested that the inhibition of the Syk gene prevents mast cell activation, and therefore, it may be an important therapeutic strategy for the treatment of allergic nasal polys^29^. Furthermore, the Wnt signaling pathway is another intracellular signal transduction that has been suggested to contribute in the pathogenesis of NPs. A recent study found that the expression levels of some Wnt signaling pathway-related genes are dysregulated in CRSwNPs, and also such a pathway is activated^30^. Another study suggested that the Wnt signaling pathway may contribute to the pathogenesis of NPs through epithelial-to-mesenchymal transition^24^. In addition, some studies have suggested that p38 Mitogen-activated protein kinase (MAPK) and Jun N-terminal kinase (JNK) signal transduction pathways are involved in CRSwNP by mediating the expression of glucocorticoid receptor isoforms^26^. Finally, MUC1, a transmembrane protein that plays a role in intracellular signaling, acts as an anti-inflammatory molecule in some airway infections and mediates the expression of anti-inflammatory genes in CRS^31^. Taken together, the role of “intracellular signal transduction” in the pathogenesis of CRSwNPs is sensible.

The “arachidonic acid” pathway is an intricate metabolic pathway that constitutes a complex biochemical cascade resulting in the synthesis of pro-inflammatory eicosanoids, including prostaglandins, thromboxanes, and leukotrienes^32,33^. Arachidonic acid and its metabolites have received considerable interest in the context of the pathogenesis of various inflammatory diseases, including CRSwNP^34–36^. According to a recent study, the severity of chronic rhinosinusitis (CRS) is associated with the levels of eicosanoids, arachidonic acid-derived lipid mediators, in nasal polyp secretions^37^. Another study has indicated that the expression levels of CXCL1 and CXCL8 genes, which are regulated by thromboxane A2, are up-regulated in the CRSsNP mucosa compared to controls^38^. Additionally, a previous study suggested that prostaglandin E2 was elevated in CRSwNP patients, especially in polyps from aspirin-sensitive individuals^36^. Therefore, substantial evidence suggests that the “arachidonic acid” pathway may well contribute to the pathogenesis of CRSwNP.

Although it may not seem likely at first glance, there is an association between the “spermatogenesis” pathway and nasal polyp pathogenesis; previous studies have established such a relationship. Young's syndrome, also named sinusitis-infertility syndrome, is a rare and inherited syndrome characterized by chronic sinusitis, bronchiectasis, and decreased fertility due to azoospermia. Various studies have suggested that mutation in the *Cftr* gene may be involved in the pathogenesis of CRS as well as Young's syndrome^39–42^. Furthermore, a previous study suggested that the up-regulation of a member of matrix metalloproteinase, namely disintegrin and metalloproteinase (ADAM)-33 protein, is associated with the pathogenesis of nasal polyps^43^. On the other hand, another study has indicated that such protein is expressed in spermatogenesis cells and plays a central role during sperm maturation^44,45^. Overall, the above studies demonstrate the existence of common genes in spermatogenesis process and nasal polyp pathogenesis.

The “protein metabolic processes” refer to the various pathways involved in synthesizing, breaking down, and modifying proteins. These processes are tightly regulated to ensure proper protein homeostasis and cellular function, Evidence suggests that “protein metabolic processes” may be disrupted in nasal polyp disease, leading to abnormal protein synthesis and degradation. A transcriptome study of nasal epithelial cells from patients with Anti-Inflammatory Drug-Exacerbated Respiratory Disease (AERD) indicated that the leukotrienes metabolic process actively participates in such disease^46^. Furthermore, proteome studies of nasal mucosa from healthy and CRS patients have shown that the disruption of, either positive or negative, regulation of cellular protein metabolic processes can be involved in the pathogenesis of CRS^47,48^.

The biological relevancy of the four triplets was confirmed using both GSEA and GRN (see Fig. 3). Moreover, the importance of two switch genes, *S100a9* and *Tgfbr3*, belonging to such triplets was confirmed using Random Forest Analysis (see Fig. 5). Accordingly, it is suggested that these triplets may play a central role in CRSwNP.

We investigated the biological relationship among genes involved in above mentioned triplets in the literature. A biological relevancy in the *S100a9*/{*Cd14*, *Tpd52l1*} triplet was found. We discuss this triplets in more detail.

### The relationships among involved genes in *S100a9*/{*Cd14*, *Tpd52l1*} triplet

The *S100a9*/{*Cd14*, *Tpd52l1*} triplet was selected to be explained in more detail because it includes several features that distinguish it from other triplets. First, this triple is not only statistically significant but also is biologically relevant based on both GSEA and GRN. Second, according to Random Forest analysis, the expression of *S100a9* gene is significantly different in NPs patients (Fig. 5). Finally, we could find significant evidence in the literature to confirm such relations.

However, to the best of our knowledge, no major evidence found for three others suggested triplets in the literature, these triplets should be studied further.

In the following, we discussed the importance of *S100a9*, *Cd14* and *Tpd52l1* genes in CRSwNP. See below.

The *S100a9* gene encodes a calcium-binding protein called *S100a9*, a member of the S100 protein family. This gene is predominantly expressed by myeloid cells, including neutrophils and monocytes, and plays a role in the regulation of inflammation and immune responses^49^. Crombruggen and colleagues showed that the protein expression level of S100A9 was significantly increased in CRSwNP patients, resulting in increased deposition on extracellular matrix (ECM) structures of CRSwNP tissue compared to CRS without NP and controls. It suggested that the inflammatory/remodeling, as the major characteristic of CRSwNP, empowers the retention of S100A9 protein in the ECM CRSwNP tissue. In this way, the S100A9 protein acts as a local danger signal-inducing inflammatory mediator, predominantly via TLR-4 activation^50^. Brouk and colleagues showed that the expression level of *S100a9* is evaluated in CRSwNP at both mRNA and protein levels. Furthermore, they indicated that such a phenomenon coincides with increased matrix metalloproteinases (MMPs) production in CRSwNP, implying the contribution of *S100a9* and MMPs in elevated nasal cell proliferation^51^. Moreover, the up-regulation of *S100a9* expression in the nasal polyp tissues of patients with CRSwNP compared to the inferior turbinate tissue of both healthy control and CRS without NP patients was reported in recent studies^52,53^.

The *Cd14* gene encodes a glycosylphosphatidylinositol-anchored membrane protein expressed on various immune cells’ surfaces, including monocytes, macrophages, and dendritic cells. The *Cd14* acts as a co-receptor for lipopolysaccharide (LPS), a component of the outer membrane of Gram-negative bacteria. Its principal function is facilitating the recognition and binding of LPS by Toll-like receptor 4 (TLR4), initiating an inflammatory response. Several studies have investigated the role of the *Cd14* gene in CRSwNP pathogenesis. Yazdani and colleagues investigated genetic variations in the *Cd14* gene and their association with CRSwNP susceptibility. They suggested that specific single nucleotide polymorphisms (SNPs) in the *Cd14* promoter gene might be associated with NP pathogenesis and asthma incidence^17^. Yao and colleagues suggested that *Cd14*, as an inflammatory mediator, is significantly up-regulated in noneosinophilic CRSwNP as compared to eosinophilic CRSwNP and controls^54^. These findings could provide further insights regarding the diverse nature of CRSwNP. Furthermore, CD14 is known as a marker for monocyte-derived dendritic cells (moDCs). O'Connell and colleagues demonstrated that CRSwNP is distinguished by a significant increase in circulating moDCs, associated with systemic inflammation exhibiting a Th2 bias as well as mucosal inflammation^55^.

T*pd52l1*, also known as Tumor protein D52-like 1, is a member of the TPD52 family of proteins. It has been implicated in various cellular processes, including cell proliferation, apoptosis, and vesicular trafficking^56^. The role of *Tpd52l1* has been extensively studied in breast cancer^57^, childhood leukemia^58^, colorectal cancer^59^ and recurrent implantation failure^60^, while its role in CRSwNP pathogenesis has not been reported yet.

In the current study, the expression level of *S100a9*, as a switch gene of *S100a9*/{*Cd14*, *Tpd52l1*}, was up-regulated in NP-NP samples (see Fig. 5), in agreement with previous studies mentioned earlier. Such a gene controls the co-expression relationship between *Cd14* and *Tpd52l1* gene pairs. As shown in Fig. 4, when the normalized expression level of the *S100a9* gene is between − 2.34 and − 0.42 (as in most CS-IT samples (see Fig. 5)), there is a direct correlation between *Cd14* and *Tpd52l1* expression levels (r_low_ = 0.70). In contrast, when the normalized expression level of the *S100a9* gene is between 0.42 and 2.34 (as in most NP-NP samples (see Fig. 5)), there is an inverse correlation between *Cd14* and *Tpd52l1* expression levels (r_high_ = − 0.65).

To our knowledge, the switch role of the *S100a9* gene for the *Cd14* and *Tpd52l1* gene pair has not been reported. However, the coordination in the changes of expression level of such genes has been reported in previous studies. See below.

It has been reported that *Cd14* and *Tpd52l1* genes are up-regulated in primary lung cancer compared to the adjacent normal tissue. Interestingly, under treatment with Romidepsin, expression levels of both these genes are down-regulated^61^. Similarly, the expression levels of such genes are down-regulated under treatment with Triglitazone in stomach cell cancers^62^, and Emodlin in Hepatocellular Carcinoma^63^. Furthermore, the up-regulation of gene expression levels of both such genes has been reported in some chronic diseases, including Type 2 Diabetes Mellitus^64^ and chronic obstructive pulmonary disease^65^.

### On the role of toll-like receptor 4 signal transduction pathway in CRSwNPs

The results of GSEA suggested that the *S100a9*/{*Cd14*, *Tpd52l1*} triplet participates in the “positive regulation of intracellular signal transduction” pathway (Fig. 3A). On the other hand, an in-depth review of literature indicated that the *S100a9*, *Cd14* and *Tpd52l1* genes may be involved in CRSwNP through the toll-like receptor 4 (TLR4) signal transduction pathway, which is surprisingly consistent with our results of GSEA. See below.

The term “Positive regulation of intracellular signal transduction” refers to the biological processes that enhance or amplify signal transmission from cell surface receptors to intracellular targets. This amplification facilitates specific biochemical cascades that alter cellular functions. For instance, the activation of TLR4 by its ligands, such as LPS, triggers a cascade of intracellular signaling that leads to the transcriptional activation of genes involved in immune and inflammatory responses^66^. Some central intracellular signaling pathways positively regulated by TLR4 include the NF-κB^67^, MAPK^68^, IRF3^69^ and PI3K-Akt^70^ pathways. Additionally, according to data in the Gene Ontology (GO) database, the term “positive regulation of intracellular signal transduction” [GO:1902533] is linked to “positive regulation of the toll signaling pathway” [GO:004572] through the “positive regulation of signal transduction” [GO:009967] term^71^.

In the following, we discuss the role of TLR4 in CRSwNP, and then the role of the *S100a9*, *Cd14* and *Tpd52l1* genes in the TLR4 pathway.

Toll-like receptors (TLRs) represent transmembrane receptors characterized by two domains: an extracellular domain responsible for pathogen interaction, coupled with an intracellular signaling domain. These receptors play a central role in triggering inflammatory responses against invading microorganisms through detecting pathogen-associated molecular patterns (PAMPs) associated with foreign pathogens such as bacteria, viruses, and fungi. Specifically, TLR4 is known as a receptor for lipopolysaccharide (LPS), a PAMP found in the outer membrane of gram-negative bacteria as a main pathogenic factor in NP^72^. Some evidence suggest that the TLR4 signaling pathway is involved in the pathogenesis of CRSwNP by remodeling of nasal polyp. Cho and colleagues suggested that high expression levels of the TLR4 gene induce MAPK and PI3K/Akt signaling pathways, contributing to nasal polyps remodeling^73^. Other studies have reported that steroids inhibit the remodeling of airways in NP by decreasing the expression levels of TLR4 in both mRNA and protein levels. Indeed, expression levels of vascular endothelial growth factor (VEGF) as an airway remodeling factor in NPs can be efficiently inhibited via TLR4/Akt/NF-κB signaling pathway^74,75^.

Previous studies indicated that *S100a9* induces the NF-κB activation through the binding to the TLR4, which in turn triggers pro-inflammatory cytokine response in monocytes^76–78^. In addition, the protein levels of S100A9 is significantly increased in ECM structures of CRSwNP patients compared to CRS without NP and control ones. It has been suggested that extracellular S100A9 proteins induce the release of diverse inflammatory mediators through TLR-4 engagement^50^. Ehrchen et al. have been suggested that S100A8/ S100A9 complex can act as endogenous activators of TLR4 and promote lethal, endotoxin-induced shock^79^. Riva et al. indicated that *S100a9* acts as a TLR4 agonist and induces nuclear factor-ĸB responses^76^.

The above studies indicated that the *S100a9* gene has been shown to modulate TLR4 signaling in certain contexts. Interestingly, such a modulator role is in accordance with the concept of a switch gene.

It has been indicated that *Cd14* plays various functional roles in LPS-induced TLR4 activation. First, as the most important role, *Cd14* prompts the internalization of the LPS/TLR4 complex into endosomes^80^, where TLR4 engages cytosolic TRIF-TRAF3 signaling to stimulate IFNβ response^81^. Second, the *Cd14* binds and transfers LPS to the TLR4-MD2 complex as a long-established role, triggering the myddosome assembly and signal transduction^82^. Finally, the CD14 protein acts as an essential co-receptor for *S100a9*-mediated TLR4-stimulation^83^. Interestingly, the cooperation between *Cd14* and *S100a9* in the TLR4 signal transduction pathway is consistent with the results of the current study.

A recent study suggested that expression of *Tpd52* gene at the protein level is significantly increased in LPS-stimulated macrophage cells. In such a process, TLR4 is activated by LPS and induces the MyD88 pathway, which subsequently produces pro-inflammatory cytokines through activation of transcriptional nuclear factor (NF)-κB. Furthermore, it was indicated that the expression levels of *Tpd52* gene are significantly decreased upon treatment by statin as an anti-inflammatory agent^84^.

Although the above evidence emphasizes the role of *S100a9*, *Cd14* and *Tpd52l1* genes in the pathogenesis of CRSwNP through the TLR4 signal transduction pathway, a conclusive association is yet to be found between the expression level of such genes and the progression of CRswNP.

The two investigational drugs, Paquinimod and Tasquinimod, are quinoline-3-carboxamide derivatives known for their potential to inhibit *S100a9* biological function^85^. Although their mechanisms of such an action have not yet been fully understood, an explanation is that these drugs can directly bind to *S100a9*, preventing formation of *S100a9–S100a8* complex, and its further interaction with TLR4 and the receptor for advanced glycation end-products^86,87^. The blockage of *S100a9*-mediated pathways by these drugs leads to several immune modulatory and tumor microenvironment impairing effects; the reduced production of the key pro-inflammatory cytokines and chemokines for immune cell activation and recruitment to inflammation or tumor growth sites^88^, anti-angiogenic effects (caused by Tasquinimod)^87^, and the diminished myeloid-derived suppressor cells and tumor-associated macrophages, which can suppress the development and metastasis of tumor^88,89^. Paquinimod has been under clinical trials for the treatment of autoimmune disorders, including systemic lupus erythematosus and systemic sclerosis^90,91^. Furthermore, Tasquinimod has been studied as an immunotherapeutic agent in solid tumors, especially prostate cancer^92^, and hematological malignancies like multiple myeloma^93^ and Myelofibrosis^94^. Studies are going on to confirm the use of these investigational medications in practice. However, to the best of our knowledge, the applicability of these drugs in the treatment of CRSwNP has not been scientifically reported so far.

## Conclusion and further work

Advances in producing “omics” disease-related datasets have provided worthwhile research opportunities about disease-related pathways and genes. In this study, the three-way interaction approach was utilized for the first time to trace critical biomarkers and dysregulated biological pathways involved in CRSwNP pathogenesis. The three-way interaction approach can cope with the dynamic nature of co-expression relations by introducing a switch gene as a surrogate for the intrinsic state variable in the cell. Hence, such an approach describes a more comprehensive and precise comprehension of the underlying reasons for cellular changes. Moreover, the switch genes as the controller of the evolution in gene interactions can be considered potential therapeutic targets. Our study results revealed four dysregulated pathways in CRSwNP, including “positive regulation of intracellular signal transduction”, “arachidonic acid metabolic process”, “spermatogenesis” and “negative regulation of cellular protein metabolic process”. Additionally, the *S100a9* as a switch gene, together with the gene pair {*Cd14*, *Tpd52l1*} form a statistically significant and biologically relevant triplet. More specifically, we suggested that *S100a9* might act as a potential upstream modulator in the TLR4 transduction pathway in the major CRSwNP pathologies. Moreover, drug exploring results suggested that “Tasquinimod” and “Paquinimod” can potentially influence CRSwNP treatment by modulating the *S100a9* expression profile.

While our study provides new clues into the pathogenesis of CRSwNPs through computational approaches, further trials are required to validate such findings. In the next step, it is imperative to experimentally confirm the relationship between the *S100a9* gene and the {*Cd14*, *Tpd52l1*} gene pair.

## Methods

### Gene expression profiling dataset

The dataset used in this study comprises gene expression data from various samples. Specifically, it includes 42 samples of chronic rhinosinusitis with nasal polyps (CRSwNP-NP), 33 paired non-polyp inferior turbinate samples (CRSwNP-IT), and 28 samples from inferior turbinate controls without chronic rhinosinusitis (CS-IT). The gene expression data which was generated by the Illumina HiSeq 4000 platform is accessible in the Gene Expression Omnibus (GEO) database under the accession number GSE136825^9^.

To ensure data comparability and reliability, the raw RNA-Seq expression profiles underwent normalization using the reads per kilobase million (RPKM) values. This normalization method was implemented using the edgeR R package^95^. After normalization, 6018 genes were selected for further investigation and analysis.

Gene expression data were analyzed using one-way Analysis of Variance (ANOVA)^96^ to determine the significance of differences among the study groups. Upon establishing significant differences from the ANOVA, post-hoc comparisons between group means were conducted using Tukey’s Honest Significant Difference (HSD) test^97^. This test was chosen with the objective of comparing multiple groups simultaneously. Statistical significance was set at a p-value of less than 0.05.

### Liquid association analysis

The liquid association measure was calculated to capture three-way interactions among all genes in the dataset. It was accomplished using the fastMLA R package^14^, which employs a modified liquid association algorithm. The algorithm assesses changes in co-expression relationships between two genes, X and Y, based on the expression level of a third gene, Z.

Specifically, the fast modified liquid association algorithm assigns an MLA score to each gene triplet, quantifying the magnitude of the liquid association. In detail, MLA (Z/{X, Y}) can be estimated using the following formula:

$$\widehat{MLA}=\frac{{\sum }_{i}^{M}\widehat{{\rho }_{i}} \overline{{Z }_{i}}}{M}$$

Here, *M* represents the number of bins over Z $\widehat{{\rho }_{i}}$ is the Pearson’s correlation coefficient of X and Y in samples of the *i*th bin, and $\overline{{Z }_{i}}$ is the mean expression value of Z in the *i*th bin.

Before running the fastMLA algorithm, two preprocessing steps are required. Firstly, the marginal distribution of each variable should follow a normal distribution, as determined by Li’s approach^11^. Secondly, each variable should be standardized to have a mean of 0 and a variance of 1, which was achieved using the CTT package^15^. The first preprocessing step was performed using an in-house implementation, while the second one used the CTT package^98^.

To establish more conservative significance thresholds, considering the large number of tests conducted, the corrected p-values were estimated using the Bonferroni method^99^. Liquid association triplets with a corrected p-value less than 0.001 were deemed statistically significant. These thresholds were applied to ensure the reliable identification of significant associations amidst multiple tests.

### Functional enrichment analysis

Functional enrichment analysis, also called gene set enrichment analysis (GSEA), is a valuable statistical method used to classify genes or proteins over-represented within a specific gene set based on predefined annotations^100^. In the context of this study, the GSEA was employed for two purposes; (i) to trace the biologically relevant triplets and (ii) to identify key biological processes and pathways associated with the pathogenesis of CRSwNP in both NP and IT tissue.

This analysis focused on “biological processes” defined in the gene ontology (GO) database^71^, as well as “pathways” from the KEGG database^101^. Moreover, it was performed using the ClueGO tool^102^ within the Cytoscape environment^103^. The right-sided hypergeometric test (with a Kappa threshold of 0.4) and the Benjamini–Hochberg (BH) correction method were used for the validation of enrichment^104^.

### Gene regulatory network construction

A gene regulatory network (GRN) is employed to conceptualize intricate regulatory mechanisms that control gene expression levels within cells. It is represented as a direct graph, composed of nodes (genes) and directed edges (regulatory connections) that exhibit either activatory or inhibitory interactions. Such a graph enables the prediction of gene expression patterns in various conditions^105^.

The GRN was reconstructed on the geWorkbench (genomics Workbench) platform^106^ utilizing ARACNE (Algorithm for the Reconstruction of Accurate Cellular Networks)^107^. This algorithm employs gene expression data to reconstruct a cellular network based on a reverse engineering approach. It captures directed regulatory relationships between each transcriptional regulator and its potential target genes based on mutual information measures. Furthermore, this network was reconstructed for every gene implicated in the statistically significant triplets by considering Bonferroni-corrected p-value < 0.05^99^.

### Random forests classification

The Random Forest (RF) is an advanced classification method based on machine learning principles. It involves constructing a collection of independent decision trees using techniques like bagging and feature randomness. Each decision tree recursively partitions the data into more homogeneous subsets based on specific features, ultimately leading to accurate combined classification outputs.

A random forest classifier was constructed using the randomForest R package^108^. The algorithm was configured with a specific set of parameters. The “number of decision trees” parameter was set to 10,000 trees, ensuring a large ensemble to capture complex patterns and improve overall performance. The “mtry” parameter, determining the number of randomly selected features considered at each node, was set to the square root of the total number of features. This choice helps maintain diversity among the trees and prevent overfitting^109^. Furthermore, the gene importance measure is calculated by assessing the average increase in error rate across all the trees when a specific gene is randomly permuted.

A receiver operating characteristic (ROC) curve was generated^110^ to assess the classification performance of the random forest classifier.

### Putative drugs exploring

This study employed two prominent drug databases, DrugBank^111^ and DGIdp^112^, to identify potential drugs. DrugBank is a comprehensive and extensively curated database that provides detailed information about various drugs, including their chemical structures, pharmacological activities, mechanisms of action, and therapeutic indications. On the other hand, DGIdb is a specialized database that focuses on drug-gene interactions. It provides a comprehensive collection of genes and their drug interactions, including information on drug targets, mechanisms of action, and therapeutic associations.

### Supplementary Information

Supplementary Information.

## Data Availability

The datasets generated and/or analysed during the current study are available in the Gene Expression Omnibus (GEO) repository [GSE136825] and in the Supplementary Information files.
