# Supplementary material for: S100a9 might act as a modulator of the Toll-like receptor 4 transduction pathway in chronic rhinosinusitis with nasal polyps
Source: Sci Rep. 2024 Apr 27;14:9722. doi: 10.1038/s41598-024-60205-4 (PMC11055867; doi:10.1038/s41598-024-60205-4)
Supplement: Supplementary file 1 — Supplementary Information. [file 41598_2024_60205_MOESM1_ESM.pdf]

Supplemental Information

Table of contents

Results, Statistically significant three-way interaction determination

Fig S1……………………………………………………………………………………….2

Text S1……………………………………………………………………………………...3

Table S1…………………………………………………………………………………….3

Results, Biologically relevant three-way interaction identification

Table S2…………………………………………………………………………………..23

Results, Random Forest for Selecting Genes

Fig S3……………………………………………………………………………………..24

Table S4…………………………………………………………………………….…..26

Table S5…………………………………………………………………………………..27

Table S6…………………………………………………………………………………..28

Table S7…………………………………………………………………………………..29

References: ……………………………………………………………………………31

Results, Statistically significant three-way interaction determination

Fig S1.The p-value histogram of the top 200000 three-way interactions.

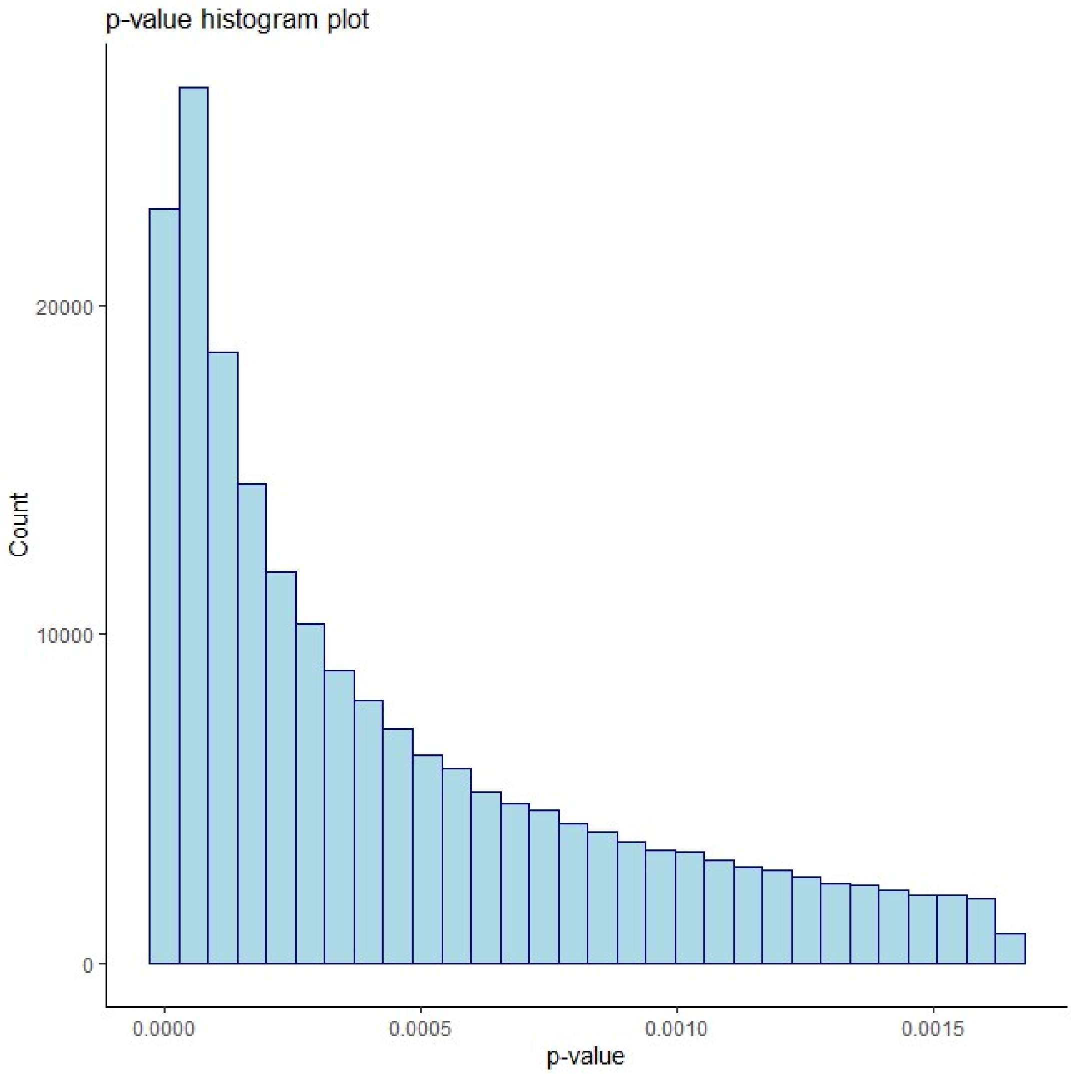
p-value histogram plot
Count
20000
10000
0
0.0000
0.0005
0.0010
0.0015
p-value

Text S1. An explanation about the chosen cut-off point

It should be noted that if we change the cut-off to be more restricted, the number of statistically significant triplets be reduced (e.g., by changing the threshold to adjusted p-value < 10^{-2}, only 127 statistically significant triplets is remained). Since it is tried to trace the biologically-relevant triplets among statistically significant ones, a shrink output may lead to miss detection of numerous biologically-relevant triplets. Therefore, according to previous studies, we considered the cut-off 0.001 can be appropriate for Liquid-association analysis [1].

Table S1. The list of 807 statistically significant triplets.

num	X	Y	Z	rhodiff	MLA value	wald	p value	bonferroni
1	ESRRG	OSBPL3	RNF227	1.3573	0.4837	54.3313	1.69E-13	2.54E-08
2	LYPD6B	PKM	MAL2	-1.4536	-0.5191	53.5229	2.56E-13	3.84E-08
3	TNFRSF19	REC8	TGFBR3	-1.3451	-0.4731	52.7591	3.77E-13	5.66E-08
4	EZR	PLEKHB1	SYNE1	1.3591	0.4827	51.4583	7.31E-13	1.10E-07
5	VWA3A	GNA15	BNIPL	-1.3407	-0.4769	50.611	1.13E-12	1.70E-07
6	KLHL6	TSPAN6	PDLIM1	1.286	0.4707	49.8026	1.70E-12	2.55E-07
7	ESRRG	OSBPL3	REC8	1.3806	0.4869	48.2311	3.79E-12	5.69E-07
8	CFAP74	GNA15	BNIPL	-1.3191	-0.4707	47.5688	5.31E-12	7.97E-07
9	INPP4B	UNC5B	KIF22	-1.4804	-0.528	45.7235	1.36E-11	2.04E-06
10	SFN	NME7	AGRN	-1.3423	-0.485	45.3211	1.67E-11	2.51E-06
11	REEP4	LRRC27	PPL	-1.3322	-0.4643	45.309	1.68E-11	2.52E-06
12	TOGARAM2	GNA15	BNIPL	-1.3672	-0.4894	45.0022	1.97E-11	2.96E-06
13	FHL2	UCP2	AGRN	-1.3276	-0.4765	44.9928	1.98E-11	2.97E-06
14	FRMPD2	SHANK2	NAA40	1.3875	0.499	44.4262	2.64E-11	3.96E-06
15	CAPN13	ADH7	PGM2L1	-1.4241	-0.5085	44.3917	2.69E-11	4.04E-06
16	CD38	ANKRD18B	SHROOM3	1.2988	0.4647	43.7279	3.77E-11	5.66E-06
17	GJB4	WLS	LRRC4	-1.3184	-0.4724	43.7083	3.81E-11	5.72E-06
18	FHL2	SYT17	AGRN	-1.3578	-0.4846	43.6088	4.01E-11	6.02E-06
19	GJB3	WLS	LRRC4	-1.4551	-0.5208	43.5357	4.16E-11	6.24E-06
20	AKR1C2	BCO2	HES2	-1.4132	-0.4867	43.0971	5.21E-11	7.82E-06
21	EZR	PRNP	MYO1B	-1.3078	-0.466	43.087	5.24E-11	7.86E-06
22	RGS12	SHROOM3	CD38	1.3161	0.4706	42.9158	5.71E-11	8.57E-06
23	EZR	CDC25B	SNED1	-1.3612	-0.4865	42.764	6.18E-11	9.27E-06
24	AK7	SLFN13	ALCAM	1.2615	0.4708	42.6368	6.59E-11	9.89E-06
25	TP63	LY6E	HES2	-1.3065	-0.4688	42.6085	6.69E-11	1.00E-05
26	EDARADD	SLC16A3	OXGR1	1.4553	0.5226	42.3907	7.47E-11	1.12E-05
27	PTPN6	ALDH3A2	MFNG	-1.3804	-0.4828	42.3852	7.50E-11	1.13E-05
28	ACOT7	JHY	AGRN	-1.3198	-0.4692	42.3413	7.67E-11	1.15E-05
29	VSNL1	GRAMD2A	CDCP1	-1.403	-0.503	42.1621	8.40E-11	1.26E-05
30	S100A16	STING1	ALDH1A1	-1.3882	-0.4983	42.1592	8.41E-11	1.26E-05
31	SHANK2	VWA3A	NAA40	1.4568	0.5234	42.1557	8.43E-11	1.26E-05

32	NCALD	ALDH3B1	NOS2	1.3146	0.4641	41.9763	9.24E-11	1.39E-05
33	DRC1	GNA15	BNIPL	-1.3333	-0.4738	41.8785	9.71E-11	1.46E-05
34	DIXDC1	TSPAN19	ALCAM	1.2757	0.4665	41.8674	9.77E-11	1.47E-05
35	MAPK10	PLEKHB1	ELK3	1.3158	0.4764	41.7464	1.04E-10	1.56E-05
36	SHANK2	LRRC74B	NAA40	1.4183	0.5101	41.6465	1.09E-10	1.64E-05
37	GNA15	FAM227A	BNIPL	-1.3075	-0.4655	41.627	1.10E-10	1.65E-05
38	DCDC1	GNA15	BNIPL	-1.3068	-0.4668	41.6123	1.11E-10	1.67E-05
39	FHL2	IQCG	AGRN	-1.2895	-0.465	41.5437	1.15E-10	1.73E-05
40	NUCB2	PLEKHG7	GPR137B	1.3009	0.467	41.5431	1.15E-10	1.73E-05
41	NUCB2	CLUAP1	ADAM12	1.34	0.4807	41.4976	1.18E-10	1.77E-05
42	ANKRD18A	TNFRSF19	TGFBR3	-1.3066	-0.4677	41.4052	1.24E-10	1.86E-05
43	PTGER2	SEMA4B	LPAR5	-1.3367	-0.4765	41.3452	1.28E-10	1.92E-05
44	WLS	CLDN8	STRBP	1.3647	0.5174	41.3015	1.30E-10	1.95E-05
45	S100A16	STING1	CLIC6	-1.3124	-0.4713	41.2934	1.31E-10	1.97E-05
46	PLA2R1	ABCC5	ATP10B	1.3449	0.4796	41.2632	1.33E-10	2.00E-05
47	CAPN13	ADH7	JPT2	-1.2858	-0.4633	41.2229	1.36E-10	2.04E-05
48	OSBPL6	JPT1	PLXNB2	-1.3424	-0.469	41.1485	1.41E-10	2.12E-05
49	FAM166B	SHANK2	NAA40	1.3966	0.5014	40.986	1.53E-10	2.30E-05
50	MSH3	TIAM1	MAL2	-1.4399	-0.5197	40.9225	1.58E-10	2.37E-05
51	ADCY2	CCL28	SYNM	-1.3846	-0.48	40.8146	1.67E-10	2.51E-05
52	SHANK2	CFAP52	NAA40	1.4198	0.5125	40.7384	1.74E-10	2.61E-05
53	ESRRG	OSBPL3	TTLL5	1.4207	0.5049	40.7086	1.77E-10	2.66E-05
54	CDC14A	MDFI	PNPLA7	1.3147	0.4762	40.6798	1.79E-10	2.69E-05
55	CD38	IQANK1	SHROOM3	1.3108	0.4695	40.6762	1.80E-10	2.70E-05
56	MCFD2	COBL	ATP2B4	-1.328	-0.466	40.6688	1.80E-10	2.70E-05
57	OSBPL6	GLRB	PLXNB2	1.4082	0.5073	40.6493	1.82E-10	2.73E-05
58	SLC2A1	CLIC5	IL4R	-1.298	-0.4634	40.5867	1.88E-10	2.82E-05
59	CFAP57	ESRRG	NDUFA4L2	1.3132	0.4667	40.5024	1.96E-10	2.94E-05
60	B3GNT2	ALPK1	SLC25A5	-1.2763	-0.4718	40.4716	1.99E-10	2.99E-05
61	ESRRG	FER1L5	LIMCH1	-1.396	-0.4938	40.4622	2.00E-10	3.00E-05
62	ESRRG	OSBPL3	LRRC46	1.3618	0.4838	40.4388	2.03E-10	3.05E-05
63	OSBPL6	NECTIN1	LRGUK	-1.3271	-0.4778	40.329	2.15E-10	3.23E-05
64	GJB3	CLUAP1	PPL	-1.3183	-0.4634	40.2719	2.21E-10	3.32E-05
65	ST6GAL2	PSCA	NDUFA4L2	-1.2986	-0.4677	40.2688	2.21E-10	3.32E-05
66	ABCC5	VNN2	FIGN	1.3552	0.485	40.263	2.22E-10	3.33E-05
67	UBXN11	GNA15	BNIPL	-1.4177	-0.5049	40.2375	2.25E-10	3.38E-05
68	FHL2	ZMYND10	AGRN	-1.3493	-0.4865	40.2356	2.25E-10	3.38E-05
69	ESRRG	OSBPL3	C21orf58	1.408	0.4952	40.2122	2.28E-10	3.42E-05
70	HACD4	CLEC2B	PPL	-1.3319	-0.4808	40.1844	2.31E-10	3.47E-05
71	INPP4B	CSTB	KIF22	-1.3823	-0.4919	40.0738	2.45E-10	3.68E-05
72	ARHGEF38	TXLNB	LMO7	1.2852	0.4666	40.0336	2.50E-10	3.75E-05

73	RCC1	APOBEC3G	LPAR5	-1.3137	-0.4664	39.9994	2.54E-10	3.81E-05
74	SGPP2	C12orf76	ANXA3	1.3086	0.4746	39.9767	2.57E-10	3.86E-05
75	NCALD	ALDH3B1	DEGS2	1.3651	0.4926	39.9676	2.58E-10	3.87E-05
76	VWA7	CDH3	SVIP	1.3671	0.4938	39.9515	2.60E-10	3.90E-05
77	NUCB2	PLEKHG7	MVB12B	1.3566	0.4731	39.9494	2.61E-10	3.92E-05
78	SFN	NME7	POLR2A	-1.3623	-0.4791	39.942	2.62E-10	3.93E-05
79	KIAA2012	GNA15	BNIPL	-1.3223	-0.4702	39.9199	2.65E-10	3.98E-05
80	PTPN13	ANKFN1	PPL	-1.3672	-0.4848	39.9075	2.66E-10	3.99E-05
81	SFN	SEMA3C	USP22	-1.2928	-0.4776	39.8505	2.74E-10	4.11E-05
82	VAV3	WDR38	PNPLA7	1.3505	0.4753	39.8177	2.79E-10	4.19E-05
83	GJB3	CDC14A	SVIP	1.3958	0.4941	39.7224	2.93E-10	4.40E-05
84	MUC2	LYZ	HSPH1	1.3017	0.4708	39.7192	2.93E-10	4.40E-05
85	SHROOM3	ROPN1L	DLK2	1.2943	0.4642	39.6818	2.99E-10	4.49E-05
86	PPOX	FGFR3	TK1	-1.2995	-0.462	39.6815	2.99E-10	4.49E-05
87	OSBPL3	SLFN13	PTGER2	1.4076	0.521	39.669	3.01E-10	4.52E-05
88	OXR1	BCO2	SDC1	1.3729	0.4855	39.6136	3.10E-10	4.65E-05
89	CFAP52	GNA15	BNIPL	-1.307	-0.4651	39.6012	3.11E-10	4.67E-05
90	CHN2	CDC45	BNIPL	-1.3645	-0.4887	39.5961	3.12E-10	4.68E-05
91	SH3BGRL2	CLUAP1	SLPI	-1.3343	-0.4766	39.5895	3.13E-10	4.70E-05
92	KNDC1	PKM	SVIP	1.3031	0.4655	39.5295	3.23E-10	4.85E-05
93	RAB38	GRAMD2A	CDCP1	-1.2965	-0.4658	39.4929	3.29E-10	4.94E-05
94	CD14	CRISP2	CARD8	-1.3136	-0.4732	39.4605	3.35E-10	5.03E-05
95	ESRRG	CLUAP1	MED25	1.3908	0.4912	39.332	3.58E-10	5.37E-05
96	LYPD6B	PKM	KRT19	-1.3708	-0.4902	39.3051	3.62E-10	5.43E-05
97	FHL2	CCDC78	AGRN	-1.309	-0.4739	39.2598	3.71E-10	5.57E-05
98	TEKT4	TNFRSF19	AK4	-1.3289	-0.4782	39.2538	3.72E-10	5.58E-05
99	CFAP299	SHANK2	PLXNB2	1.3929	0.4849	39.218	3.79E-10	5.69E-05
100	RFX3	PKM	SVIP	1.3844	0.4788	39.1141	4.00E-10	6.00E-05
101	CREB3L4	DHRS9	RNASEH2A	1.3908	0.5032	39.0886	4.05E-10	6.08E-05
102	TCTN1	FAM83D	GJB2	-1.4321	-0.5048	39.087	4.05E-10	6.08E-05
103	CHN2	MKI67	BNIPL	-1.4466	-0.5248	39.0751	4.08E-10	6.12E-05
104	TNFRSF19	TSNAXIP1	APOL1	1.31	0.4678	39.0576	4.11E-10	6.17E-05
105	CAPN13	ADH7	SELENOM	1.2827	0.4675	39.0134	4.21E-10	6.32E-05
106	S100A9	ITGA6	SMAD3	1.4252	0.5135	38.9912	4.26E-10	6.39E-05
107	EZR	PLEKHB1	ELK3	1.3757	0.4982	38.9382	4.37E-10	6.56E-05
108	ZBBX	GNA15	BNIPL	-1.3394	-0.4766	38.9337	4.38E-10	6.57E-05
109	PLEKHG7	TNFRSF19	STAT6	1.322	0.4699	38.9263	4.40E-10	6.60E-05
110	KIAA2012	SHANK2	AK4	-1.4116	-0.504	38.9038	4.45E-10	6.68E-05
111	POLQ	NUCB2	PTGFR	1.3841	0.4871	38.8806	4.51E-10	6.77E-05
112	COL7A1	GLIPR2	GAPDH	-1.3955	-0.5031	38.8751	4.52E-10	6.78E-05
113	OSBPL3	CEP41	RBM38	1.3585	0.4778	38.8674	4.54E-10	6.81E-05

114	RSPH4A	CEP41	HDGF	1.3368	0.4802	38.8604	4.55E-10	6.83E-05
115	NME9	GAS8	ANKRD65	1.3728	0.4867	38.8222	4.64E-10	6.96E-05
116	CFAP57	PKM	SVIP	1.3322	0.4773	38.795	4.71E-10	7.07E-05
117	ARL4C	SMAD3	TYMS	-1.2462	-0.4677	38.7874	4.73E-10	7.10E-05
118	TMPRSS4	RASA3	RAP2B	-1.3282	-0.4808	38.7473	4.82E-10	7.23E-05
119	CYB561A3	IL4R	LGALS3	-1.3303	-0.4694	38.7454	4.83E-10	7.25E-05
120	CCDC113	SLFN13	NUDC	1.3266	0.4766	38.6455	5.08E-10	7.62E-05
121	ABRAXAS1	DMD	ICMT	1.2996	0.4771	38.6343	5.11E-10	7.67E-05
122	ESRRG	AGBL2	VNN2	1.3528	0.4825	38.5559	5.32E-10	7.98E-05
123	SFN	BCO2	HES2	-1.4379	-0.4853	38.4926	5.50E-10	8.25E-05
124	TNFRSF19	C21orf58	APOL1	1.2957	0.466	38.492	5.50E-10	8.25E-05
125	NME7	TNFRSF19	TGFBR3	-1.3891	-0.4826	38.4729	5.55E-10	8.33E-05
126	REEP4	ANKFN1	PPL	-1.3717	-0.4899	38.4632	5.58E-10	8.37E-05
127	LAMC2	PLEKHB1	SYNE1	1.387	0.4916	38.4123	5.73E-10	8.60E-05
128	CFAP100	SHANK2	LIG1	1.3754	0.4857	38.3796	5.82E-10	8.73E-05
129	DLEC1	PKM	SVIP	1.3511	0.4694	38.3646	5.87E-10	8.81E-05
130	ESRRG	OSBPL3	WDR49	1.3858	0.4857	38.3369	5.95E-10	8.93E-05
131	CD38	PRSS12	NUCB2	1.351	0.4898	38.3262	5.99E-10	8.99E-05
132	PDE6B	GLRB	TSPAN12	-1.3035	-0.4728	38.2961	6.08E-10	9.12E-05
133	ARHGEF38	TRIP13	CTSS	1.3581	0.4892	38.2759	6.14E-10	9.21E-05
134	ESRRG	ALDH3B1	LRRN1	1.4472	0.5181	38.2544	6.21E-10	9.32E-05
135	CEP41	SAXO2	HDGF	1.2931	0.4682	38.251	6.22E-10	9.33E-05
136	SHANK2	DRC7	NAA40	1.3532	0.4868	38.2396	6.26E-10	9.39E-05
137	ESRRG	RSPH3	ACTG2	-1.3405	-0.4782	38.2196	6.32E-10	9.48E-05
138	GJB3	TOGARAM2	PPL	-1.3326	-0.4667	38.2021	6.38E-10	9.57E-05
139	MASTL	ZNF702P	HOPX	-1.3003	-0.4649	38.1858	6.43E-10	9.65E-05
140	HES2	CFP	MASTL	-1.3212	-0.4726	38.1592	6.52E-10	9.78E-05
141	P2RY2	POU2AF1	NCALD	1.3516	0.4875	38.152	6.54E-10	9.81E-05
142	FLACC1	COL7A1	GAPDH	-1.3552	-0.4865	38.1039	6.71E-10	1.01E-04
143	FHL2	DNAH9	AGRN	-1.2953	-0.4722	38.102	6.71E-10	1.01E-04
144	LEF1	JPT1	ALDH1L1	1.3212	0.4839	38.0657	6.84E-10	1.03E-04
145	ARRB2	MISP	CD200R1	-1.3183	-0.4682	38.0525	6.89E-10	1.03E-04
146	ESRRG	CFAP70	ACTG2	-1.3267	-0.4753	38.0238	6.99E-10	1.05E-04
147	GNA15	FUZ	BNIPL	-1.3379	-0.4756	37.9869	7.12E-10	1.07E-04
148	PTPN13	HACD4	PPL	-1.3103	-0.4634	37.9642	7.21E-10	1.08E-04
149	EFCAB12	PKM	SVIP	1.3157	0.4731	37.9509	7.25E-10	1.09E-04
150	CEP41	RAB36	AK4	-1.4629	-0.5197	37.9284	7.34E-10	1.10E-04
151	CCDC39	MYOF	SVIP	1.321	0.4677	37.8838	7.51E-10	1.13E-04
152	SHANK2	TTLL9	PLXNB2	1.388	0.483	37.8728	7.55E-10	1.13E-04
153	KLHL6	PRDM1	LRRC4	-1.3589	-0.4858	37.8671	7.57E-10	1.14E-04
154	KRT14	RIPK4	S100A9	1.3182	0.4719	37.805	7.82E-10	1.17E-04

155	TRIM3	ALDH3B1	PLXNB2	1.3524	0.4736	37.7877	7.89E-10	1.18E-04
156	CLEC10A	TOP2A	TMEM45B	-1.2947	-0.4669	37.7668	7.97E-10	1.20E-04
157	GALNT14	SDK1	MCM4	-1.304	-0.4669	37.6748	8.36E-10	1.25E-04
158	GNA15	KCNE1	BNIPL	-1.3034	-0.4649	37.6441	8.49E-10	1.27E-04
159	NCALD	MOK	CLIC1	1.3029	0.4706	37.638	8.52E-10	1.28E-04
160	ARHGEF4	SHROOM3	CD38	1.2898	0.4687	37.6369	8.52E-10	1.28E-04
161	CXCL6	TNC	MEIS1	1.4008	0.5096	37.6336	8.54E-10	1.28E-04
162	SHANK2	CCDC78	NAA40	1.4136	0.5073	37.6292	8.56E-10	1.28E-04
163	LAPTM5	CD14	SLC16A3	1.3052	0.4644	37.6251	8.57E-10	1.29E-04
164	TNFRSF18	TPD52L1	BRI3BP	1.3139	0.4655	37.6161	8.61E-10	1.29E-04
165	CFAP100	SHANK2	AK4	-1.4088	-0.5007	37.6048	8.66E-10	1.30E-04
166	MORN1	NCALD	MED25	1.326	0.464	37.5845	8.75E-10	1.31E-04
167	GALNT14	AGBL2	NT5C	-1.3496	-0.4773	37.5839	8.76E-10	1.31E-04
168	ARHGEF38	DNAH11	LIG1	1.317	0.4678	37.5817	8.77E-10	1.32E-04
169	ALPL	IFITM1	CFAP20	-1.2829	-0.468	37.5527	8.90E-10	1.34E-04
170	ESRRG	DNAH11	EGLN3	1.3012	0.4736	37.5413	8.95E-10	1.34E-04
171	SORBS2	IP6K3	PLK1	1.3355	0.4646	37.5192	9.05E-10	1.36E-04
172	ESRRG	OSBPL3	LRRIQ1	1.3545	0.4783	37.4606	9.33E-10	1.40E-04
173	CFAP299	CDH3	SVIP	1.3212	0.4666	37.4545	9.36E-10	1.40E-04
174	CYP26A1	GJB2	TCTN1	-1.3567	-0.4894	37.4282	9.48E-10	1.42E-04
175	ESRRG	DNAH3	CLPTM1L	-1.3199	-0.4746	37.4216	9.52E-10	1.43E-04
176	GALNT14	UCP2	STMN1	-1.3833	-0.49	37.4041	9.60E-10	1.44E-04
177	CREG1	ATP5F1D	ABHD12	-1.319	-0.4727	37.4028	9.61E-10	1.44E-04
178	C1orf87	TMPRSS4	PLXNB2	-1.3227	-0.4676	37.3942	9.65E-10	1.45E-04
179	C1orf87	NCALD	AK4	-1.3826	-0.492	37.3847	9.70E-10	1.46E-04
180	CD200R1	AZGP1	F2RL1	1.3196	0.4704	37.3782	9.73E-10	1.46E-04
181	AK8	CDH3	SVIP	1.3123	0.4712	37.3618	9.81E-10	1.47E-04
182	NQO1	PRNP	CYSTM1	-1.312	-0.4681	37.3598	9.82E-10	1.47E-04
183	TOGARAM2	SHANK2	PLXNB2	1.3606	0.4781	37.3429	9.91E-10	1.49E-04
184	SPAG17	TNFRSF19	AK4	-1.3484	-0.4816	37.3401	9.92E-10	1.49E-04
185	ESRRG	OSBPL3	NPHP1	1.3406	0.4761	37.3309	9.97E-10	1.50E-04
186	MCM4	RAB27B	FAT2	1.3439	0.4782	37.2787	1.02E-09	1.53E-04
187	SERPINI2	GNA15	BNIPL	-1.3286	-0.4725	37.2654	1.03E-09	1.55E-04
188	NCALD	VWA3A	NAA40	1.3029	0.4649	37.2636	1.03E-09	1.55E-04
189	OSBPL6	HS3ST1	ARL3	-1.3315	-0.4727	37.216	1.06E-09	1.59E-04
190	NEK5	PKM	SVIP	1.3101	0.473	37.1959	1.07E-09	1.61E-04
191	EPHA4	CYP2F1	MAP9	-1.3504	-0.4803	37.1868	1.07E-09	1.61E-04
192	SYNE1	PLEKHB1	CAPN5	1.3312	0.4717	37.1813	1.08E-09	1.62E-04
193	SLC6A15	PSENEN	STAT6	-1.2897	-0.475	37.1506	1.09E-09	1.64E-04
194	ESRRG	OSBPL3	DNAH12	1.3178	0.4723	37.113	1.11E-09	1.67E-04
195	FHL2	MS4A8	AGRN	-1.284	-0.4659	37.0797	1.13E-09	1.70E-04

196	CNGA4	GNA15	BNIPL	-1.333	-0.4754	37.0727	1.14E-09	1.71E-04
197	SLC22A4	SHANK2	CAPN5	1.4426	0.5174	37.0713	1.14E-09	1.71E-04
198	IL5RA	GNA15	BNIPL	-1.3534	-0.4805	37.0617	1.14E-09	1.71E-04
199	TRIM3	PLEKHG7	BAIAP2	1.2706	0.4664	37.0615	1.14E-09	1.71E-04
200	WLS	ESRRG	SMAD9	-1.2948	-0.4648	37.0507	1.15E-09	1.73E-04
201	DNAH5	GNA15	BNIPL	-1.3107	-0.4678	37.0072	1.18E-09	1.77E-04
202	VWA7	PKM	SVIP	1.33	0.4827	37.0042	1.18E-09	1.77E-04
203	SHANK2	KIF19	NAA40	1.3804	0.496	36.9822	1.19E-09	1.79E-04
204	GJB3	EFHB	PPL	-1.35	-0.473	36.9649	1.20E-09	1.80E-04
205	WLS	RAB38	MISP	-1.2827	-0.464	36.9624	1.20E-09	1.80E-04
206	ESRRG	OSBPL3	ZNF487	1.3607	0.4895	36.9538	1.21E-09	1.82E-04
207	OSBPL6	JPT1	MUC4	-1.344	-0.4781	36.9335	1.22E-09	1.83E-04
208	CCDC30	PTPN13	IQANK1	-1.3066	-0.4689	36.9331	1.22E-09	1.83E-04
209	C2orf73	TIMELESS	SLC41A2	1.2889	0.4693	36.9125	1.24E-09	1.86E-04
210	REEP1	UCP2	DUOX1	1.3382	0.4819	36.8932	1.25E-09	1.88E-04
211	SHANK2	DNAAF6	PLXNB2	1.339	0.4662	36.8864	1.25E-09	1.88E-04
212	ESRRG	OSBPL3	LINC00271	1.3459	0.474	36.869	1.26E-09	1.89E-04
213	CFAP65	SHANK2	NAA40	1.3722	0.4935	36.8677	1.26E-09	1.89E-04
214	PLEKHG7	JPT1	FDXR	-1.3582	-0.4798	36.8654	1.27E-09	1.91E-04
215	NCALD	C11orf49	AK4	-1.3468	-0.4807	36.862	1.27E-09	1.91E-04
216	TTC29	CLEC2B	AGRN	-1.3117	-0.4698	36.8459	1.28E-09	1.92E-04
217	VNN1	WDR86	PIR	-1.3413	-0.4758	36.8408	1.28E-09	1.92E-04
218	MAL2	PEAK1	CLEC7A	-1.3507	-0.4768	36.8318	1.29E-09	1.94E-04
219	SYNE1	ITPR2	NUCB2	-1.4311	-0.5148	36.8087	1.30E-09	1.95E-04
220	PTPN13	NLRC3	PPL	-1.4557	-0.5172	36.8081	1.30E-09	1.95E-04
221	C1orf87	SHANK2	PLXNB2	1.3642	0.4752	36.8026	1.31E-09	1.97E-04
222	KIF19	GNA15	BNIPL	-1.3103	-0.4671	36.7976	1.31E-09	1.97E-04
223	PPP1R14C	NCALD	REC8	1.3752	0.4878	36.7863	1.32E-09	1.98E-04
224	DCDC1	P2RY2	REEP4	-1.3441	-0.4757	36.7819	1.32E-09	1.98E-04
225	TEKT4	SERPINB4	BAK1	-1.3413	-0.4778	36.7424	1.35E-09	2.03E-04
226	SHISA5	C10orf67	SVIP	1.3355	0.4855	36.731	1.36E-09	2.04E-04
227	LRRC56	CDH3	SVIP	1.3069	0.4666	36.7271	1.36E-09	2.04E-04
228	FHL2	TTC29	AGRN	-1.3176	-0.4755	36.7053	1.37E-09	2.06E-04
229	VAV3	IGFBP2	PNPLA7	1.3762	0.484	36.6984	1.38E-09	2.07E-04
230	CFAP91	CFAP43	AGR3	1.3162	0.4739	36.6754	1.40E-09	2.10E-04
231	TNFRSF19	KIF19	NOSTRIN	-1.4065	-0.501	36.67	1.40E-09	2.10E-04
232	FHL2	RUVBL1	POLR2A	-1.3373	-0.4682	36.6542	1.41E-09	2.12E-04
233	SPAG8	SHANK2	PLXNB2	1.3594	0.4733	36.6131	1.44E-09	2.16E-04
234	PTPN13	TSNAXIP1	CELSR2	-1.3258	-0.4718	36.6033	1.45E-09	2.18E-04
235	GCNT3	CSTB	B3GNT2	1.3755	0.491	36.6004	1.45E-09	2.18E-04
236	FHL2	DYDC2	AGRN	-1.3668	-0.4937	36.5868	1.46E-09	2.19E-04

237	CCDC30	PKM	SVIP	1.3399	0.4696	36.5734	1.47E-09	2.21E-04
238	GJB3	NLRC3	IL4R	-1.3108	-0.4699	36.5723	1.47E-09	2.21E-04
239	TNFAIP8L1	TSPAN6	PACRG	1.306	0.4691	36.5555	1.48E-09	2.22E-04
240	TOGARAM2	TNFRSF19	AK4	-1.3823	-0.494	36.5548	1.48E-09	2.22E-04
241	FHAD1	SHANK2	NAA40	1.3959	0.5013	36.5488	1.49E-09	2.24E-04
242	SHROOM3	LRRIQ1	DLK2	1.3132	0.4709	36.5486	1.49E-09	2.24E-04
243	CFAP157	SHANK2	NAA40	1.3841	0.4998	36.548	1.49E-09	2.24E-04
244	ESRRG	OSBPL3	SAXO2	1.313	0.4642	36.5306	1.50E-09	2.25E-04
245	DNAI1	SHANK2	AK4	-1.3756	-0.4889	36.4907	1.53E-09	2.30E-04
246	SHANK2	VWA3A	AK4	-1.3674	-0.4873	36.4825	1.54E-09	2.31E-04
247	ALDH3B1	SHANK2	PPP6R1	1.4586	0.5188	36.4701	1.55E-09	2.33E-04
248	SLC16A1	DHRS9	MGAM2	1.3094	0.467	36.4605	1.56E-09	2.34E-04
249	VDR	SLC6A16	SHANK2	1.3562	0.4857	36.4482	1.57E-09	2.36E-04
250	GSDMC	SMPD3	PPL	-1.3201	-0.4634	36.433	1.58E-09	2.37E-04
251	CDC14A	ESRRG	ODAM	-1.5558	-0.5574	36.4313	1.58E-09	2.37E-04
252	FHL2	CIBAR2	AGRN	-1.3003	-0.47	36.4154	1.59E-09	2.39E-04
253	GJB3	SUPT7L	IGFBP3	-1.3215	-0.4677	36.4054	1.60E-09	2.40E-04
254	DNAH5	SHANK2	NAA40	1.3292	0.4781	36.388	1.62E-09	2.43E-04
255	ABRAXAS1	NCALD	ICMT	1.3692	0.4965	36.3808	1.62E-09	2.43E-04
256	S100A9	RPGR	LRP1	-1.3117	-0.4725	36.3741	1.63E-09	2.45E-04
257	IK	GM2A	SVIP	1.3345	0.4724	36.3438	1.65E-09	2.48E-04
258	CRACR2A	PLEKHG7	PTHLH	1.2964	0.4731	36.3431	1.65E-09	2.48E-04
259	CCDC78	GNA15	BNIPL	-1.3072	-0.4658	36.3373	1.66E-09	2.49E-04
260	PRR5L	P2RY2	CRYBG1	-1.3177	-0.466	36.3362	1.66E-09	2.49E-04
261	SRPRA	TROAP	DHRS3	1.3554	0.4856	36.3298	1.67E-09	2.51E-04
262	ESRRG	AGBL2	CLPTM1L	-1.3142	-0.4719	36.3229	1.67E-09	2.51E-04
263	CLEC10A	KPNA2	CLDN7	-1.3995	-0.493	36.2988	1.69E-09	2.54E-04
264	KLHL6	TSPAN6	CFAP36	1.3878	0.5012	36.297	1.69E-09	2.54E-04
265	OSBPL6	HS3ST1	SOD1	-1.2832	-0.4615	36.2913	1.70E-09	2.55E-04
266	ALDH3B1	TYROBP	ADGRE5	-1.3192	-0.4708	36.2796	1.71E-09	2.57E-04
267	TTC16	TRIM3	PLXNB1	1.3446	0.4834	36.2668	1.72E-09	2.58E-04
268	GNA14	JPT1	WASF1	-1.375	-0.491	36.2507	1.73E-09	2.60E-04
269	SHISA5	TSPAN19	AGRN	-1.3335	-0.4768	36.2411	1.74E-09	2.61E-04
270	WNT9A	TNFRSF19	DSC2	-1.3195	-0.4675	36.2389	1.75E-09	2.63E-04
271	ANKRD66	NUCB2	VNN2	1.2975	0.4692	36.2354	1.75E-09	2.63E-04
272	SYK	ALDH3A2	TPD52L1	1.3424	0.4787	36.2044	1.78E-09	2.67E-04
273	ZBBX	SHANK2	PLXNB2	1.3989	0.4911	36.1904	1.79E-09	2.69E-04
274	STING1	JPT1	GNA15	-1.5133	-0.5426	36.1884	1.79E-09	2.69E-04
275	SPAG6	GNA15	BNIPL	-1.3106	-0.4671	36.1847	1.79E-09	2.69E-04
276	GJB2	TTLL9	FAM83D	-1.3316	-0.4742	36.1734	1.81E-09	2.72E-04
277	FHL2	ENKUR	RNF207	-1.3584	-0.4803	36.168	1.81E-09	2.72E-04

278	SPAG6	TNFRSF19	APOL1	1.3156	0.468	36.1653	1.81E-09	2.72E-04
279	LRP2BP	PKP3	BCL11B	-1.3294	-0.4614	36.164	1.81E-09	2.72E-04
280	OSBPL6	NCALD	HILPDA	1.3343	0.4837	36.1607	1.82E-09	2.73E-04
281	S100A9	LRRC18	ABCC5	-1.3598	-0.4936	36.1489	1.83E-09	2.75E-04
282	CCDC17	SHANK2	PLXNB2	1.4118	0.4955	36.133	1.84E-09	2.76E-04
283	GJB3	RFX3	PPL	-1.3755	-0.4865	36.1215	1.85E-09	2.78E-04
284	C7orf57	TNFRSF19	APOL1	1.3198	0.4696	36.0996	1.87E-09	2.81E-04
285	NCALD	IQCH	MED25	1.3137	0.4744	36.0931	1.88E-09	2.82E-04
286	TTC23L	REEP4	PPL	-1.4017	-0.5087	36.0912	1.88E-09	2.82E-04
287	ARMC3	GNA15	BNIPL	-1.3196	-0.4696	36.0815	1.89E-09	2.84E-04
288	ECM1	DNAJA4	S100A6	-1.3155	-0.4689	36.0785	1.90E-09	2.85E-04
289	FHL2	TMEM154	IL4R	-1.2946	-0.4636	36.0593	1.91E-09	2.87E-04
290	RP1	SHANK2	AK4	-1.3859	-0.495	36.0553	1.92E-09	2.88E-04
291	UPK1B	GCNT3	LDHD	-1.3099	-0.4619	36.0416	1.93E-09	2.90E-04
292	TCEA3	LYPD6B	GPX1	1.3861	0.4946	36.0407	1.93E-09	2.90E-04
293	EZR	PLEKHB1	APBB1	1.299	0.4777	36.0316	1.94E-09	2.91E-04
294	SFN	LRRC49	AGRN	-1.2996	-0.466	36.0242	1.95E-09	2.93E-04
295	WDR49	MDFI	PNPLA7	1.3306	0.4833	36.0241	1.95E-09	2.93E-04
296	ALDH3B1	PLEKHB1	ELK3	1.4518	0.5218	36.0232	1.95E-09	2.93E-04
297	KIAA2012	PKM	SVIP	1.4187	0.5052	36.0222	1.95E-09	2.93E-04
298	WWC1	C22orf15	NOS2	1.2698	0.4703	36.0216	1.95E-09	2.93E-04
299	DZIP1L	PKM	SVIP	1.3243	0.474	36.0172	1.96E-09	2.94E-04
300	WNT9A	DIXDC1	KRT14	-1.3239	-0.4706	36.0164	1.96E-09	2.94E-04
301	FLACC1	SHANK2	PLXNB2	1.364	0.4721	36.012	1.96E-09	2.94E-04
302	ESRRG	DNAH3	FICD	-1.3628	-0.4896	36.0008	1.97E-09	2.96E-04
303	ESRRG	ZNF440	PAFAH1B3	1.3277	0.4763	36.0007	1.97E-09	2.96E-04
304	UPK1B	CLMN	GJB2	-1.4472	-0.5125	35.9999	1.97E-09	2.96E-04
305	STING1	PPIF	UPK1B	-1.3367	-0.4767	35.9978	1.98E-09	2.97E-04
306	MYOF	GCNT3	TMEM45B	1.3396	0.4775	35.9898	1.98E-09	2.97E-04
307	CFAP70	MYOF	SVIP	1.3592	0.4804	35.9823	1.99E-09	2.99E-04
308	TSPAN8	RAB37	MAL2	1.346	0.4778	35.9771	2.00E-09	3.00E-04
309	FER1L5	TNFRSF19	TGFBR3	-1.3594	-0.4774	35.9769	2.00E-09	3.00E-04
310	TPD52L1	IL18	ATP10B	1.3138	0.4649	35.9615	2.01E-09	3.02E-04
311	BDH1	TUBA1A	SPEF2	1.3659	0.4835	35.9613	2.01E-09	3.02E-04
312	BCO2	ALDH6A1	SDC1	1.3209	0.4737	35.9504	2.02E-09	3.03E-04
313	SLC2A1	REC8	SVIP	1.3206	0.4665	35.9477	2.03E-09	3.05E-04
314	TOGARAM2	SHANK2	LIG1	1.2982	0.4636	35.9461	2.03E-09	3.05E-04
315	COL7A1	GLIPR2	PLP2	-1.3553	-0.4886	35.9433	2.03E-09	3.05E-04
316	H2BC21	CLDN3	TOB1	-1.3542	-0.4843	35.9362	2.04E-09	3.06E-04
317	GJB3	SAXO2	PPL	-1.347	-0.4728	35.9313	2.04E-09	3.06E-04
318	MUC15	DYRK1B	CCDC66	1.3338	0.48	35.9131	2.06E-09	3.09E-04

319	AGBL2	PKM	SVIP	1.3963	0.4987	35.8983	2.08E-09	3.12E-04
320	PLEKHB1	TUBA1A	RAET1E	1.3546	0.4688	35.8962	2.08E-09	3.12E-04
321	RAB3B	PTPN13	RGS12	-1.3051	-0.4668	35.894	2.08E-09	3.12E-04
322	RGS22	TMPRSS4	PLXNB2	-1.3575	-0.4782	35.8898	2.09E-09	3.14E-04
323	UBXN11	TNFRSF19	TGFBR3	-1.394	-0.4836	35.8784	2.10E-09	3.15E-04
324	CDKN1A	STRA6	PTGS2	-1.33	-0.4654	35.8745	2.10E-09	3.15E-04
325	TGFBI	ADORA2B	EFHD2	-1.3798	-0.4898	35.8677	2.11E-09	3.17E-04
326	TMEM40	MFNG	LRP6	1.3089	0.4657	35.8604	2.12E-09	3.18E-04
327	CAPN13	ADH7	SEMA3C	-1.4735	-0.5317	35.8476	2.13E-09	3.20E-04
328	CXCL6	NOS2	PPP1R9A	1.468	0.5187	35.8385	2.14E-09	3.21E-04
329	GPX2	PTPRT	SLC16A1	-1.3276	-0.4795	35.8383	2.14E-09	3.21E-04
330	SH3BGRL2	ENKUR	SLPI	-1.3571	-0.4782	35.8366	2.15E-09	3.23E-04
331	LRRC10B	SHANK2	PLXNB2	1.3793	0.4844	35.8324	2.15E-09	3.23E-04
332	ARRB2	BCAS4	RGS14	-1.2998	-0.4704	35.8297	2.15E-09	3.23E-04
333	ADH7	GJB2	CFAP57	-1.3381	-0.4708	35.8247	2.16E-09	3.24E-04
334	GJB3	SLC27A2	MVP	-1.3366	-0.4753	35.8174	2.17E-09	3.26E-04
335	SFN	DNALI1	AGRN	-1.3	-0.467	35.8134	2.17E-09	3.26E-04
336	NEK10	CEP41	AK4	-1.331	-0.4701	35.8116	2.17E-09	3.26E-04
337	GJB3	KIF27	PPL	-1.3537	-0.4848	35.8112	2.17E-09	3.26E-04
338	CFAP70	PKM	SVIP	1.4541	0.5152	35.8109	2.17E-09	3.26E-04
339	GJB3	IFT140	POLA2	-1.2917	-0.467	35.8039	2.18E-09	3.27E-04
340	CCDC181	TMPRSS4	PLXNB2	-1.3466	-0.4757	35.8012	2.19E-09	3.29E-04
341	ESRRG	CCDC89	CLPTM1L	-1.3035	-0.4695	35.7931	2.19E-09	3.29E-04
342	P4HA1	RAB37	AHSA1	-1.2963	-0.4628	35.7857	2.20E-09	3.30E-04
343	GJB3	IFT172	TK1	-1.3346	-0.4752	35.7797	2.21E-09	3.32E-04
344	GSDME	TEX9	PHLDA3	-1.3339	-0.4758	35.7776	2.21E-09	3.32E-04
345	DCDC1	SHANK2	NAA40	1.2937	0.4654	35.7682	2.22E-09	3.33E-04
346	RASAL1	STARD13	SHANK2	1.2851	0.4654	35.7636	2.23E-09	3.35E-04
347	ANKDD1B	GNA15	BNIPL	-1.3103	-0.4672	35.7558	2.24E-09	3.36E-04
348	TOGARAM2	SHANK2	SRGAP3	1.3423	0.477	35.7528	2.24E-09	3.36E-04
349	EYA4	BIN2	PDE4D	1.354	0.4817	35.7509	2.24E-09	3.36E-04
350	IFT74	TNFRSF19	TGFBR3	-1.4636	-0.5178	35.7506	2.24E-09	3.36E-04
351	GLRB	BCO2	LTB4R	1.3557	0.4797	35.7498	2.24E-09	3.36E-04
352	UBXN11	GJB3	PPL	-1.3775	-0.4833	35.7275	2.27E-09	3.41E-04
353	REEP4	CFAP47	PPL	-1.336	-0.4629	35.7267	2.27E-09	3.41E-04
354	LRRC61	PKM	ELF5	1.371	0.4842	35.7195	2.28E-09	3.42E-04
355	GBP6	ENO2	CORO1C	-1.3568	-0.4808	35.719	2.28E-09	3.42E-04
356	COBL	SVEP1	RHOBTB3	-1.305	-0.4736	35.7178	2.28E-09	3.42E-04
357	SHANK2	TTLL9	NAA40	1.4004	0.5036	35.7169	2.28E-09	3.42E-04
358	MORN1	SH3BGRL2	SLPI	-1.3697	-0.4845	35.7119	2.29E-09	3.44E-04
359	CC2D2A	TNFRSF19	PLXNB2	1.3245	0.4623	35.6962	2.31E-09	3.47E-04

360	PKM	KIF19	SVIP	1.4487	0.5167	35.6917	2.31E-09	3.47E-04
361	GBP6	CLEC10A	SYK	-1.3412	-0.4842	35.6907	2.31E-09	3.47E-04
362	RSPH4A	TNFRSF19	AK4	-1.3405	-0.48	35.6764	2.33E-09	3.50E-04
363	CFAP65	TNFRSF19	AK4	-1.3115	-0.4686	35.6717	2.34E-09	3.51E-04
364	PLEKHG7	SERPINB4	LRRC4	-1.3522	-0.4834	35.6707	2.34E-09	3.51E-04
365	H2BC21	FHL2	OBSCN	-1.3672	-0.4861	35.6637	2.34E-09	3.51E-04
366	CFAP299	PKM	SVIP	1.4254	0.5054	35.6457	2.37E-09	3.56E-04
367	SHANK2	LRRC46	NAA40	1.3962	0.5031	35.641	2.37E-09	3.56E-04
368	COL7A1	HACD4	GAPDH	-1.3682	-0.4902	35.6314	2.38E-09	3.57E-04
369	PKM	ANKFN1	SVIP	1.3446	0.4871	35.6217	2.40E-09	3.60E-04
370	CCDC153	PKM	SVIP	1.3745	0.4841	35.6152	2.40E-09	3.60E-04
371	NSG1	C11orf49	SLC20A2	-1.3001	-0.4652	35.6085	2.41E-09	3.62E-04
372	FOCAD	ZNF385A	GSDMC	-1.3961	-0.5042	35.6072	2.41E-09	3.62E-04
373	EFHC1	REEP4	PPL	-1.3457	-0.4684	35.5915	2.43E-09	3.65E-04
374	RFX3	MYOF	SVIP	1.343	0.4696	35.5877	2.44E-09	3.66E-04
375	IL5RA	SHANK2	PLXNB2	1.344	0.4704	35.5875	2.44E-09	3.66E-04
376	SHROOM3	CAPSL	DLK2	1.3046	0.4674	35.5729	2.46E-09	3.69E-04
377	PKM	DNAAF1	SVIP	1.3704	0.4878	35.5725	2.46E-09	3.69E-04
378	APBB1	ANXA2	CDC25B	-1.2983	-0.4659	35.5673	2.46E-09	3.69E-04
379	FHL2	IQCG	ITPR3	-1.2939	-0.4715	35.5657	2.47E-09	3.71E-04
380	HCLS1	PLAC8	TOB1	1.3518	0.4848	35.5648	2.47E-09	3.71E-04
381	DZIP1L	BTC	B9D1	1.2708	0.4613	35.5516	2.48E-09	3.72E-04
382	DNAH12	CEP41	AK4	-1.3743	-0.4875	35.5379	2.50E-09	3.75E-04
383	GJB3	HACD4	PPL	-1.3518	-0.4783	35.533	2.51E-09	3.77E-04
384	ALDH3B1	TMEM120B	PTGFR	1.3671	0.4945	35.5269	2.52E-09	3.78E-04
385	FHL2	C6orf118	AGRN	-1.3214	-0.4781	35.5117	2.54E-09	3.81E-04
386	SFN	GRAMD2A	CDCP1	-1.3205	-0.4801	35.5108	2.54E-09	3.81E-04
387	CDHR3	PKM	SVIP	1.3771	0.4887	35.5075	2.54E-09	3.81E-04
388	ITGA6	P2RY2	CRYBG1	1.4145	0.5022	35.5011	2.55E-09	3.83E-04
389	ARHGEF38	DMKN	CTSS	1.3218	0.4718	35.4996	2.55E-09	3.83E-04
390	SLC12A2	ABCC4	LAD1	1.3021	0.4684	35.4996	2.55E-09	3.83E-04
391	PHGDH	SELP	SLC9A3R1	1.3671	0.4884	35.4993	2.55E-09	3.83E-04
392	CROCC2	PKM	SVIP	1.3338	0.4722	35.4802	2.58E-09	3.87E-04
393	CDC14A	GNA15	BNIPL	-1.3231	-0.4712	35.4785	2.58E-09	3.87E-04
394	MAB21L4	IFT74	ATP1B3	-1.3811	-0.493	35.4742	2.58E-09	3.87E-04
395	ARMH1	SHANK2	DTX2	1.3842	0.4985	35.4704	2.59E-09	3.89E-04
396	EIF4EBP1	CSTB	TGFBR3	-1.4863	-0.5328	35.4637	2.60E-09	3.90E-04
397	EFHD2	GLIPR2	CTSV	-1.4581	-0.5206	35.4443	2.62E-09	3.93E-04
398	FHL2	DNAJB13	AGRN	-1.304	-0.4694	35.4357	2.64E-09	3.96E-04
399	SHANK2	DNAH3	NAA40	1.3475	0.4849	35.434	2.64E-09	3.96E-04
400	ARMH1	GALNT14	STMN1	-1.4081	-0.5085	35.43	2.64E-09	3.96E-04

401	HNF4G	GAS7	ARHGEF38	1.3461	0.475	35.4133	2.67E-09	4.01E-04
402	COL7A1	THNSL1	GAPDH	-1.2998	-0.4652	35.4073	2.67E-09	4.01E-04
403	MAP3K19	GNA15	BNIPL	-1.3183	-0.4683	35.4036	2.68E-09	4.02E-04
404	CDC14A	ESRRG	EGLN3	1.3834	0.4988	35.4029	2.68E-09	4.02E-04
405	EIF4EBP1	ALDH18A1	LDLRAD3	1.2468	0.477	35.3991	2.69E-09	4.04E-04
406	ILDR1	ALDH16A1	IL1R2	-1.3219	-0.467	35.3978	2.69E-09	4.04E-04
407	PLEKHG7	TNFRSF19	BBS12	1.5386	0.5505	35.3811	2.71E-09	4.07E-04
408	FHL2	AK7	RNF207	-1.3306	-0.4695	35.3742	2.72E-09	4.08E-04
409	ALDH3B1	GNA15	BNIPL	-1.3901	-0.4938	35.3721	2.72E-09	4.08E-04
410	CAPN13	ADH7	ABCA13	-1.4578	-0.5136	35.372	2.72E-09	4.08E-04
411	ERICH3	PTPN13	CELSR2	-1.3237	-0.4698	35.3697	2.73E-09	4.10E-04
412	SLCO2B1	KDELR3	BCL2L11	1.4816	0.5285	35.3682	2.73E-09	4.10E-04
413	SHANK2	CFAP53	PLXNB2	1.3479	0.4678	35.3634	2.74E-09	4.11E-04
414	CFAP221	SHANK2	PLXNB2	1.3762	0.4822	35.3529	2.75E-09	4.13E-04
415	ESRRG	OSBPL3	FUZ	1.4226	0.5056	35.333	2.78E-09	4.17E-04
416	FMO3	SPON2	UPK1B	-1.3655	-0.4868	35.3292	2.78E-09	4.17E-04
417	GTF2E2	GOLM1	PROS1	1.2994	0.4672	35.3235	2.79E-09	4.19E-04
418	ALPL	IFITM1	RPP38	-1.3494	-0.4837	35.3229	2.79E-09	4.19E-04
419	DAPP1	PPM1H	CDC14B	1.3326	0.4774	35.3179	2.80E-09	4.20E-04
420	GALNT14	CEP19	NT5C	-1.3265	-0.4667	35.3096	2.81E-09	4.22E-04
421	ESRRG	ATF7IP2	ACTG2	-1.3041	-0.4668	35.3026	2.82E-09	4.23E-04
422	MAP3K19	TNFRSF19	APOL1	1.3634	0.486	35.2985	2.83E-09	4.25E-04
423	SMAGP	ELK3	NCALD	1.3724	0.4923	35.2884	2.84E-09	4.26E-04
424	SRD5A2	PPP1R36	DNAJB9	-1.3303	-0.4721	35.2777	2.86E-09	4.29E-04
425	PEAK1	ISG20	MALL	-1.3192	-0.4736	35.2755	2.86E-09	4.29E-04
426	CEP41	SPAG6	C1QTNF1	-1.3532	-0.4808	35.2696	2.87E-09	4.31E-04
427	BDH1	DNAI2	NWD1	1.3284	0.479	35.26	2.88E-09	4.32E-04
428	PLAC8	MB	BAIAP3	1.4612	0.5294	35.259	2.89E-09	4.34E-04
429	RP1	SHANK2	ALOX15	1.4595	0.5239	35.2476	2.90E-09	4.35E-04
430	ARHGEF38	ABCA13	CTSS	1.3653	0.4829	35.2418	2.91E-09	4.37E-04
431	LDLRAD1	CDH3	SVIP	1.319	0.4653	35.2405	2.91E-09	4.37E-04
432	FHL2	PRR29	AGRN	-1.3571	-0.4893	35.2367	2.92E-09	4.38E-04
433	MROH9	GLRB	FIGN	-1.3504	-0.4852	35.2347	2.92E-09	4.38E-04
434	ESRRG	TOGARAM1	MYH11	-1.4287	-0.5296	35.2234	2.94E-09	4.41E-04
435	BCO2	CSTB	MEAK7	-1.3107	-0.4661	35.214	2.95E-09	4.43E-04
436	SHANK2	TEX26	PLXNB2	1.4074	0.4926	35.206	2.97E-09	4.46E-04
437	PAQR7	PGM2	ZNF273	-1.3078	-0.4679	35.2001	2.98E-09	4.47E-04
438	SHROOM3	C20orf85	DLK2	1.3109	0.4686	35.1884	2.99E-09	4.49E-04
439	ABHD12B	KCTD15	PAFAH1B3	-1.3009	-0.4672	35.174	3.02E-09	4.53E-04
440	TNFRSF19	HNF1B	AK4	-1.2977	-0.4654	35.1717	3.02E-09	4.53E-04
441	RIPOR2	DMKN	SHROOM3	1.2957	0.4687	35.1548	3.05E-09	4.58E-04

442	SMAD3	KRT14	S100A9	1.3304	0.4778	35.1546	3.05E-09	4.58E-04
443	UBXN11	SLC2A1	SVIP	1.3938	0.4838	35.1368	3.07E-09	4.61E-04
444	ESRRG	RSPH3	PLN	-1.3126	-0.4714	35.1307	3.08E-09	4.62E-04
445	KIF3B	FAM83D	MMRN1	1.3594	0.4712	35.1157	3.11E-09	4.67E-04
446	SHANK2	KCNE1	ITPR3	1.3478	0.4831	35.115	3.11E-09	4.67E-04
447	ESRRG	CDHR4	EGLN3	1.2935	0.468	35.1077	3.12E-09	4.68E-04
448	SH3BGRL2	TNFAIP8L1	SLPI	-1.4013	-0.4966	35.0891	3.15E-09	4.73E-04
449	ITGA6	ABCC5	S100A9	1.3954	0.494	35.0863	3.15E-09	4.73E-04
450	ESRRG	TNFAIP8L1	FICD	-1.4507	-0.5241	35.0835	3.16E-09	4.74E-04
451	ESRRG	OSBPL3	IQCH	1.3681	0.4752	35.081	3.16E-09	4.74E-04
452	PLEKHG7	TYROBP	ADGRE5	-1.3818	-0.4908	35.0801	3.16E-09	4.74E-04
453	SFN	IQCA1	KRT4	-1.4161	-0.4969	35.0758	3.17E-09	4.76E-04
454	EIF4EBP1	TTC9	S100A16	1.304	0.4654	35.0694	3.18E-09	4.77E-04
455	ESRRG	WDR49	MED25	1.3698	0.49	35.0603	3.20E-09	4.80E-04
456	PPP1R9A	GRAMD2A	CEACAM6	1.3162	0.4652	35.0533	3.21E-09	4.82E-04
457	GJB3	CFAP91	PPL	-1.3202	-0.4715	35.0486	3.22E-09	4.83E-04
458	IL18	RAB27B	ZWINT	1.3278	0.4695	35.0479	3.22E-09	4.83E-04
459	FCGR2B	PRSS12	POU2AF1	-1.3737	-0.4915	35.0373	3.23E-09	4.85E-04
460	ERICH3	CDH3	SVIP	1.3167	0.4689	35.0318	3.24E-09	4.86E-04
461	BEST4	NCALD	DUSP6	-1.2937	-0.471	35.0254	3.25E-09	4.88E-04
462	TPD52L1	PDE3B	PLCB4	1.3422	0.4749	35.0243	3.26E-09	4.89E-04
463	COL7A1	RP1	GAPDH	-1.3619	-0.4872	35.0192	3.26E-09	4.89E-04
464	CREG1	ATP5F1D	TNIP2	-1.3441	-0.4775	35.0188	3.27E-09	4.91E-04
465	CTNNBIP1	KRT5	LMNB1	1.2717	0.4654	35.0172	3.27E-09	4.91E-04
466	STPG1	ESRRG	MBOAT7	1.3484	0.4729	35.0168	3.27E-09	4.91E-04
467	CFAP65	SHANK2	AK4	-1.3256	-0.4717	35.0062	3.29E-09	4.94E-04
468	KLHL6	CEP41	BDKRB2	1.3458	0.4927	35.0029	3.29E-09	4.94E-04
469	RBM43	ZNF213	BCL11B	-1.3047	-0.4743	35.0024	3.29E-09	4.94E-04
470	CAPN13	ALDH3A1	PGM2L1	-1.3626	-0.4863	35.0022	3.29E-09	4.94E-04
471	TTC16	SHANK2	CAPN5	1.419	0.5083	34.9965	3.30E-09	4.95E-04
472	CFAP100	CDH3	SVIP	1.3186	0.4687	34.9954	3.30E-09	4.95E-04
473	CXCL6	NOS2	IGFBP3	-1.405	-0.4995	34.9952	3.31E-09	4.97E-04
474	GLRB	BCO2	SDC1	1.3446	0.4748	34.9875	3.32E-09	4.98E-04
475	ESRRG	SRD5A2	LRRN1	1.3885	0.4966	34.9756	3.34E-09	5.01E-04
476	IL5RA	SHROOM3	RERG	1.345	0.4774	34.9733	3.34E-09	5.01E-04
477	TNFRSF19	CFAP47	AK4	-1.3509	-0.4869	34.9731	3.34E-09	5.01E-04
478	DNAH11	REEP4	PPL	-1.3329	-0.4704	34.9676	3.35E-09	5.03E-04
479	SFN	CFAP221	JAG2	-1.2878	-0.4623	34.967	3.35E-09	5.03E-04
480	SHROOM3	FAM166B	DLK2	1.3111	0.4695	34.9578	3.37E-09	5.06E-04
481	SHROOM3	VWA3A	VWA5A	1.2902	0.4679	34.9564	3.37E-09	5.06E-04
482	EFHB	NCALD	AK4	-1.4073	-0.5009	34.9512	3.38E-09	5.07E-04

483	CLDN16	TNFRSF19	POU2AF1	1.2909	0.4778	34.9463	3.39E-09	5.09E-04
484	GALNT14	C10orf67	INKA1	-1.3933	-0.4955	34.9405	3.40E-09	5.10E-04
485	MAP3K19	SHROOM3	DLK2	1.3626	0.4885	34.9364	3.41E-09	5.12E-04
486	RGS12	CD38	CLDN3	1.4933	0.5358	34.9334	3.41E-09	5.12E-04
487	CLGN	TPD52L1	PRKX	-1.3094	-0.47	34.9327	3.41E-09	5.12E-04
488	PTGES	CYP2B6	NFE2L2	-1.3423	-0.486	34.9317	3.41E-09	5.12E-04
489	TP73	MUC15	RIPOR2	1.3071	0.4697	34.9278	3.42E-09	5.13E-04
490	PKM	CCDC33	SVIP	1.3276	0.4799	34.9132	3.45E-09	5.18E-04
491	ST8SIA4	TIAM1	TMEM45B	-1.3252	-0.4706	34.9108	3.45E-09	5.18E-04
492	ESRRG	DNAI2	EGLN3	1.2451	0.4677	34.9096	3.45E-09	5.18E-04
493	CCDC17	WIPF3	PLXNB2	1.311	0.4638	34.902	3.47E-09	5.21E-04
494	FHAD1	SHANK2	ALOX15	1.4079	0.508	34.9009	3.47E-09	5.21E-04
495	C10orf67	MFNG	SVEP1	-1.356	-0.48	34.9009	3.47E-09	5.21E-04
496	CREB3L4	LGALS9B	GLB1L	1.3148	0.4688	34.8955	3.48E-09	5.22E-04
497	UPK1B	GNMT	GCNT3	-1.2985	-0.468	34.884	3.50E-09	5.25E-04
498	MYOF	CFAP58	SVIP	1.3558	0.4783	34.8748	3.52E-09	5.28E-04
499	MUC15	DNAH2	MINDY4	1.3662	0.4777	34.8746	3.52E-09	5.28E-04
500	GJB3	USH1C	NCALD	1.3638	0.4818	34.8584	3.55E-09	5.33E-04
501	ALDH3B1	CDH3	SVIP	1.3719	0.5044	34.8506	3.56E-09	5.34E-04
502	NUCB2	TNFAIP8L1	HENMT1	1.3728	0.4976	34.8459	3.57E-09	5.36E-04
503	GJB3	TOGARAM2	SRGAP3	-1.4024	-0.4936	34.8452	3.57E-09	5.36E-04
504	SHANK2	TEX26	NAA40	1.2957	0.4642	34.8398	3.58E-09	5.37E-04
505	ESRRG	CFAP300	SCGB3A1	-1.3523	-0.4647	34.8369	3.59E-09	5.39E-04
506	ACTG1	SAMHD1	RIPK4	-1.4005	-0.4927	34.8358	3.59E-09	5.39E-04
507	STK33	TNFRSF19	AK4	-1.3648	-0.4855	34.8249	3.61E-09	5.42E-04
508	NUSAP1	CLEC10A	ITPR2	1.3099	0.4709	34.8104	3.63E-09	5.45E-04
509	CD14	SULF1	S100A9	1.3081	0.4713	34.8045	3.65E-09	5.48E-04
510	ESRRG	OSBPL3	SPACA9	1.3655	0.4794	34.7938	3.67E-09	5.51E-04
511	LZTFL1	GLRB	PPIF	1.2497	0.4678	34.7893	3.67E-09	5.51E-04
512	SHANK2	CFAP52	PLXNB2	1.4286	0.4993	34.785	3.68E-09	5.52E-04
513	MAPK10	TYROBP	ADGRE5	-1.3113	-0.4638	34.7786	3.69E-09	5.54E-04
514	CCDC30	P2RY2	REEP4	-1.328	-0.4683	34.7779	3.70E-09	5.55E-04
515	TNFRSF19	RAB36	AK4	-1.4554	-0.5201	34.7711	3.71E-09	5.57E-04
516	FRMPD2	SHANK2	AK4	-1.3298	-0.4739	34.767	3.72E-09	5.58E-04
517	UBXN11	SFN	AGRN	-1.3219	-0.4758	34.7607	3.73E-09	5.60E-04
518	EZR	PLEKHB1	SVEP1	1.3389	0.4721	34.7588	3.73E-09	5.60E-04
519	TOGARAM2	SHANK2	NAA40	1.3313	0.4788	34.7542	3.74E-09	5.61E-04
520	SHANK2	KCNE1	NAA40	1.3913	0.5006	34.7484	3.75E-09	5.63E-04
521	ELK3	KDELR3	GDF15	1.3077	0.4688	34.7406	3.77E-09	5.66E-04
522	DCDC2B	ECM1	S100A6	-1.3116	-0.4704	34.7328	3.78E-09	5.67E-04
523	PRSS12	TSPAN6	POU2AF1	1.4425	0.5506	34.7276	3.79E-09	5.69E-04

524	MCFD2	ADAP2	LRRN1	1.3135	0.4687	34.7192	3.81E-09	5.72E-04
525	BRD3OS	FAM83D	GJB2	-1.3306	-0.4664	34.7168	3.81E-09	5.72E-04
526	ANKRD45	STAT2	RHOC	-1.309	-0.4739	34.7163	3.81E-09	5.72E-04
527	PLAC8	MB	C21orf58	1.3983	0.49	34.7027	3.84E-09	5.76E-04
528	CD200R1	NKX3-1	MAL2	1.3443	0.4776	34.7005	3.85E-09	5.78E-04
529	PTPN13	SPEF2	PPL	-1.3151	-0.47	34.7002	3.85E-09	5.78E-04
530	ARHGEF38	PLEKHG7	CHN2	1.2962	0.47	34.6948	3.86E-09	5.79E-04
531	SEMA3C	SEMA4B	USP22	-1.2445	-0.4629	34.6938	3.86E-09	5.79E-04
532	ESRRG	OSBPL3	JPT2	1.3592	0.484	34.6932	3.86E-09	5.79E-04
533	MAPK15	GNA15	BNIPL	-1.3109	-0.4671	34.6923	3.86E-09	5.79E-04
534	PTGES	TENM4	ALDH1L2	1.4122	0.5102	34.6885	3.87E-09	5.81E-04
535	STING1	BAG1	ALDH1A1	-1.3974	-0.5044	34.6869	3.87E-09	5.81E-04
536	PROM1	PLEKHG7	JPT2	1.3239	0.4789	34.6868	3.87E-09	5.81E-04
537	PLAC8	PLEKHB1	ELK3	1.4092	0.495	34.6811	3.88E-09	5.82E-04
538	ESRRG	IFT88	ACTG2	-1.3296	-0.4752	34.6757	3.89E-09	5.84E-04
539	CD14	TBC1D24	MFNG	-1.3923	-0.4943	34.6737	3.90E-09	5.85E-04
540	SHANK2	CCDC78	CAPN5	1.3958	0.5015	34.6718	3.90E-09	5.85E-04
541	SHANK2	CFAP52	CAPN5	1.4171	0.508	34.6695	3.91E-09	5.87E-04
542	SHROOM3	LRRC18	DLK2	1.4049	0.5039	34.6653	3.92E-09	5.88E-04
543	DAW1	STAT2	RHOC	-1.3331	-0.4832	34.6595	3.93E-09	5.90E-04
544	SHANK2	FBXO15	ITPR3	1.3075	0.4693	34.6593	3.93E-09	5.90E-04
545	WLS	RAP2B	DYNLT1	-1.2711	-0.4707	34.658	3.93E-09	5.90E-04
546	PLEKHB1	DMKN	EML1	1.3001	0.467	34.6576	3.93E-09	5.90E-04
547	NUCB2	SAMHD1	MAL2	1.392	0.4964	34.6566	3.93E-09	5.90E-04
548	CD109	BCO2	CIB1	-1.2664	-0.4643	34.6519	3.94E-09	5.91E-04
549	TRAF3IP1	CEP41	DSC2	-1.321	-0.4691	34.6492	3.95E-09	5.93E-04
550	SHANK2	KCNE1	SRGAP3	1.3937	0.4956	34.6476	3.95E-09	5.93E-04
551	SLC16A1	BCO2	GSTP1	-1.3272	-0.4781	34.6451	3.96E-09	5.94E-04
552	KCNN3	SMAD3	PPP1R3B	1.3105	0.4722	34.6288	3.99E-09	5.99E-04
553	MTHFD1L	RASA3	CHI3L2	1.3258	0.4929	34.6276	3.99E-09	5.99E-04
554	ESRRG	WDR49	VNN2	1.3113	0.4648	34.6269	3.99E-09	5.99E-04
555	DNAI1	GNA15	BNIPL	-1.3189	-0.469	34.625	4.00E-09	6.00E-04
556	CFAP100	SHANK2	MVP	1.3341	0.4794	34.6202	4.01E-09	6.02E-04
557	TSPAN19	CLDN8	NYNRIN	1.3159	0.4718	34.618	4.01E-09	6.02E-04
558	ETV1	BARX2	TBXAS1	-1.3308	-0.471	34.6128	4.02E-09	6.03E-04
559	CEP41	CFAP46	TSGA10	1.329	0.474	34.61	4.03E-09	6.05E-04
560	MYOF	TTLL6	AGRN	-1.2884	-0.4715	34.6077	4.03E-09	6.05E-04
561	TRIB2	CYP4F3	GRAMD4	-1.4209	-0.5044	34.6046	4.04E-09	6.06E-04
562	SHROOM3	ENKUR	DLK2	1.3111	0.4701	34.6	4.05E-09	6.08E-04
563	DTHD1	PKM	SVIP	1.3152	0.4647	34.5992	4.05E-09	6.08E-04
564	BTC	ALDH3B1	TULP3	1.355	0.4871	34.5936	4.06E-09	6.09E-04

565	FHL2	GAS2L2	SVIP	1.3107	0.4693	34.5934	4.06E-09	6.09E-04
566	KIF24	TNFRSF19	LMO7	1.3023	0.4714	34.5918	4.07E-09	6.11E-04
567	HMGCS2	CD200R1	SDR16C5	1.4072	0.5048	34.5783	4.09E-09	6.14E-04
568	S100A9	PLEKHG7	HEY2	-1.3659	-0.493	34.5731	4.11E-09	6.17E-04
569	ESRRG	OSBPL3	CCDC30	1.3714	0.4941	34.5707	4.11E-09	6.17E-04
570	MYOF	CNGA4	SVIP	1.335	0.4697	34.5704	4.11E-09	6.17E-04
571	QPRT	VSIG4	SHROOM3	-1.3474	-0.4931	34.5698	4.11E-09	6.17E-04
572	CFAP74	PKM	SVIP	1.4042	0.5062	34.5617	4.13E-09	6.20E-04
573	PTPN13	DTX3	ITGB4	-1.4907	-0.5299	34.5595	4.13E-09	6.20E-04
574	FLACC1	GLRB	SCAMP4	1.3053	0.4611	34.5552	4.14E-09	6.21E-04
575	ESRRG	C10orf67	PAFAH1B3	1.3284	0.4774	34.5552	4.14E-09	6.21E-04
576	CCDC170	TNFRSF19	APOL1	1.3156	0.4673	34.555	4.14E-09	6.21E-04
577	TNFRSF19	DNAAF1	NOSTRIN	-1.3671	-0.4874	34.5499	4.15E-09	6.23E-04
578	ESRRG	KIAA2012	PAFAH1B3	1.286	0.4638	34.5397	4.18E-09	6.27E-04
579	CD14	TPD52L1	S100A9	-1.3515	-0.482	34.5388	4.18E-09	6.27E-04
580	NSG1	VNN2	CSRP1	1.3369	0.4674	34.5388	4.18E-09	6.27E-04
581	RSPH10B	GNA15	BNIPL	-1.3802	-0.4912	34.5243	4.21E-09	6.32E-04
582	CFAP100	SHANK2	LRRC45	1.3046	0.4617	34.5218	4.22E-09	6.33E-04
583	GJB3	LMNTD1	CDH3	-1.2752	-0.4648	34.518	4.22E-09	6.33E-04
584	GJB3	RSPH4A	NUAK2	-1.3457	-0.4784	34.5125	4.24E-09	6.36E-04
585	DHRS9	CLDN8	NYNRIN	1.386	0.4953	34.5033	4.26E-09	6.39E-04
586	SHANK2	HYDIN	NAA40	1.3551	0.4865	34.5026	4.26E-09	6.39E-04
587	CFAP97D2	PKM	SVIP	1.3121	0.4705	34.5021	4.26E-09	6.39E-04
588	TEKT4	PKM	SVIP	1.3663	0.4846	34.4988	4.27E-09	6.41E-04
589	SFN	DAPP1	B3GNT2	-1.3779	-0.4951	34.4835	4.30E-09	6.45E-04
590	PLB1	SLC6A15	TMEM201	-1.3407	-0.4767	34.4819	4.30E-09	6.45E-04
591	SHANK2	TLCD2	ITGA6	-1.3305	-0.4735	34.4774	4.31E-09	6.47E-04
592	ALDH3B1	GLB1L2	ERICH2	1.3118	0.4733	34.471	4.33E-09	6.50E-04
593	VNN2	FICD	PTHLH	1.3142	0.4646	34.4687	4.33E-09	6.50E-04
594	ESRRG	CAPS2	ANGPTL1	-1.4103	-0.5005	34.4679	4.33E-09	6.50E-04
595	FHL2	CFAP221	ITPR3	-1.2787	-0.4653	34.4678	4.33E-09	6.50E-04
596	DNAJB9	PPP1R36	GNA14	-1.3094	-0.47	34.465	4.34E-09	6.51E-04
597	FGFR3	EFHC1	TK1	-1.3395	-0.4771	34.4644	4.34E-09	6.51E-04
598	ARMH1	CDH3	ELF5	1.3607	0.4766	34.4589	4.35E-09	6.53E-04
599	ESRRG	OSBPL3	DZIP3	1.314	0.4734	34.4563	4.36E-09	6.54E-04
600	SH3BGRL2	ANKRD18A	VNN2	1.325	0.4737	34.4511	4.37E-09	6.56E-04
601	BCO2	NECTIN1	DTX3	-1.3351	-0.4724	34.4429	4.39E-09	6.59E-04
602	GLRB	BCO2	TFE3	1.3329	0.4725	34.4414	4.39E-09	6.59E-04
603	GALNT14	NOS2	B3GNT2	-1.384	-0.4937	34.4371	4.40E-09	6.60E-04
604	NSG1	SERPING1	PLAGL2	-1.3191	-0.4775	34.4368	4.40E-09	6.60E-04
605	GJB3	CFAP53	POLA2	-1.2585	-0.4674	34.4368	4.40E-09	6.60E-04

606	RHOC	TGM2	BRI3BP	-1.3184	-0.4739	34.4253	4.43E-09	6.65E-04
607	TPD52L1	CDK1	PLP2	1.4811	0.5283	34.4252	4.43E-09	6.65E-04
608	HCLS1	CA2	PLEKHA5	1.3064	0.4708	34.4156	4.45E-09	6.68E-04
609	SH3BGRL2	REC8	SLPI	-1.3937	-0.5005	34.4153	4.45E-09	6.68E-04
610	SHANK2	EPPIN	AK4	-1.3623	-0.4874	34.4153	4.45E-09	6.68E-04
611	NUCB2	CORO7	ITPR2	-1.346	-0.487	34.4146	4.45E-09	6.68E-04
612	ESRRG	NAT1	SCGB3A1	-1.3959	-0.4813	34.4115	4.46E-09	6.69E-04
613	SHANK2	DNAAF1	NAA40	1.3934	0.5007	34.4086	4.47E-09	6.71E-04
614	CCDC17	GNA15	BNIPL	-1.3558	-0.4827	34.4075	4.47E-09	6.71E-04
615	C10orf67	SHANK2	CAPN5	1.3748	0.4924	34.4074	4.47E-09	6.71E-04
616	ECM1	RABL2A	S100A6	-1.3084	-0.4666	34.4032	4.48E-09	6.72E-04
617	IGSF3	SLC35D2	BAIAP2	-1.3001	-0.4636	34.3979	4.49E-09	6.74E-04
618	GJB3	ACYP1	RRM2	-1.3871	-0.4902	34.3932	4.50E-09	6.75E-04
619	GJB3	PTGER2	LPAR5	-1.3823	-0.4943	34.3925	4.50E-09	6.75E-04
620	DNAH11	TNFRSF19	AK4	-1.465	-0.5242	34.3873	4.52E-09	6.78E-04
621	BCO2	JPT1	C12orf75	-1.4074	-0.5017	34.3855	4.52E-09	6.78E-04
622	SRGAP2C	SMAGP	CD36	-1.388	-0.4964	34.3829	4.53E-09	6.80E-04
623	DNAH5	PKM	SVIP	1.3901	0.4896	34.3814	4.53E-09	6.80E-04
624	CFAP74	FHL2	RNF207	-1.3126	-0.4659	34.3813	4.53E-09	6.80E-04
625	SLC4A4	AKR1C2	GALNT14	-1.3245	-0.4745	34.3778	4.54E-09	6.81E-04
626	KRT14	SERPINB13	S100A9	1.3577	0.4865	34.3691	4.56E-09	6.84E-04
627	CD14	FOXC1	CHN1	-1.3227	-0.4672	34.358	4.59E-09	6.89E-04
628	CYP4B1	SH3BGRL2	MBOAT7	1.3509	0.4741	34.3576	4.59E-09	6.89E-04
629	HCLS1	CA2	ALDH3A2	1.3984	0.4906	34.3551	4.59E-09	6.89E-04
630	THNSL1	TIAM1	MARK2	-1.3097	-0.467	34.354	4.59E-09	6.89E-04
631	CXCL6	COLCA2	CD177	-1.3463	-0.465	34.3534	4.60E-09	6.90E-04
632	CFAP46	SHANK2	NAA40	1.3199	0.4747	34.3503	4.60E-09	6.90E-04
633	C7orf57	CEP41	WDR19	1.3061	0.4674	34.35	4.60E-09	6.90E-04
634	KCNAB2	ITPR2	NPL	1.4312	0.5093	34.3413	4.62E-09	6.93E-04
635	CXCL6	PYCR1	ADGRG6	-1.3341	-0.48	34.3371	4.63E-09	6.95E-04
636	ESRRG	WDR49	CLPTM1L	-1.3645	-0.4904	34.3352	4.64E-09	6.96E-04
637	ESRRG	CFAP43	EGLN3	1.3001	0.4733	34.3253	4.66E-09	6.99E-04
638	CEP41	CIBAR2	AK4	-1.3132	-0.4699	34.3209	4.67E-09	7.01E-04
639	FHL2	LRRC46	AGRN	-1.2968	-0.4694	34.3139	4.69E-09	7.04E-04
640	VNN1	SLC41A2	PSENEN	1.2908	0.463	34.3124	4.69E-09	7.04E-04
641	UBXN11	CSTB	MAL2	-1.4168	-0.5008	34.3099	4.70E-09	7.05E-04
642	NME7	SOX15	AGRN	-1.3166	-0.4722	34.3082	4.70E-09	7.05E-04
643	CAPN13	KLK10	ARHGEF16	-1.3506	-0.4743	34.2973	4.73E-09	7.10E-04
644	DSC2	SERPINB4	ZBTB7C	1.2927	0.469	34.2966	4.73E-09	7.10E-04
645	SHROOM3	RSPH1	DLK2	1.3443	0.4816	34.2962	4.73E-09	7.10E-04
646	RAB11FIP1	NOTCH1	KDELR3	1.2919	0.4892	34.2946	4.74E-09	7.11E-04

647	DHRS9	SIGLEC10	CNTRL	-1.3956	-0.5034	34.2935	4.74E-09	7.11E-04
648	CPA3	EZR	VAV3	-1.3049	-0.4674	34.2871	4.76E-09	7.14E-04
649	NUCB2	PLEKHG7	ADAM12	1.3572	0.4878	34.2762	4.78E-09	7.17E-04
650	EFHD2	CD14	MFNG	1.3317	0.4787	34.2729	4.79E-09	7.19E-04
651	NUCB2	C22orf15	CASP8	1.3432	0.4733	34.2722	4.79E-09	7.19E-04
652	MAPK10	LILRB4	ADGRE5	-1.3699	-0.4808	34.2687	4.80E-09	7.20E-04
653	ESRRG	CLDN16	TPD52L1	-1.3193	-0.473	34.2682	4.80E-09	7.20E-04
654	CEP41	ARMC3	C1QTNF1	-1.3561	-0.482	34.2647	4.81E-09	7.22E-04
655	FHAD1	SHANK2	AK4	-1.3795	-0.4905	34.2415	4.87E-09	7.31E-04
656	GJB2	FAM83D	CLMN	-1.4413	-0.5141	34.2307	4.90E-09	7.35E-04
657	TPD52L1	CSTB	POC1A	1.3547	0.4786	34.229	4.90E-09	7.35E-04
658	ZNF473	TIAM1	B4GALT5	-1.3113	-0.471	34.2206	4.92E-09	7.38E-04
659	CCDC17	SHANK2	NAA40	1.4122	0.5083	34.2157	4.93E-09	7.40E-04
660	S100A2	IFT172	SRC	-1.3403	-0.4678	34.2104	4.95E-09	7.43E-04
661	CCDC33	CDH3	SVIP	1.3082	0.47	34.2059	4.96E-09	7.44E-04
662	PPP4R1	TGM2	FIGN	1.2922	0.4677	34.2005	4.97E-09	7.46E-04
663	RRM2	CLEC10A	GALNT16	1.3219	0.4721	34.1971	4.98E-09	7.47E-04
664	GJB3	CFAP52	NUAK2	-1.3096	-0.469	34.1954	4.98E-09	7.47E-04
665	CDC14A	ESRRG	PLN	-1.3459	-0.4831	34.1881	5.00E-09	7.50E-04
666	AGRN	AKR1C3	FBXO36	-1.3723	-0.491	34.1865	5.01E-09	7.52E-04
667	FHL2	HNF1B	POLR2A	-1.3092	-0.4611	34.1857	5.01E-09	7.52E-04
668	ESRRG	OSBPL3	POLA2	1.3217	0.4702	34.1824	5.02E-09	7.53E-04
669	ESRRG	OSBPL3	ZMYND10	1.3568	0.4768	34.18	5.02E-09	7.53E-04
670	PDE3B	TYROBP	SERPINA1	1.2847	0.4645	34.1761	5.03E-09	7.55E-04
671	LRRC71	PKM	SVIP	1.3202	0.4695	34.1739	5.04E-09	7.56E-04
672	DNAH6	PKM	SVIP	1.3157	0.4676	34.1694	5.05E-09	7.58E-04
673	CTTNBP2NL	CCDC33	SVIP	1.2928	0.4662	34.1692	5.05E-09	7.58E-04
674	SHROOM3	CIBAR2	DLK2	1.3844	0.4966	34.1648	5.06E-09	7.59E-04
675	CDHR4	SHANK2	AK4	-1.3871	-0.4946	34.1642	5.07E-09	7.61E-04
676	ABRAXAS1	REEP4	ICMT	-1.272	-0.4669	34.1634	5.07E-09	7.61E-04
677	TNFRSF19	VWA3A	SLAIN1	-1.4843	-0.5296	34.1532	5.09E-09	7.64E-04
678	TRMT1L	ESRRG	EGLN3	1.3021	0.4674	34.1497	5.10E-09	7.65E-04
679	CFAP100	TNFRSF19	C3orf70	-1.4326	-0.5102	34.1494	5.10E-09	7.65E-04
680	ELAPOR1	ISYNA1	DYNLRB1	1.3075	0.4669	34.1376	5.13E-09	7.70E-04
681	SYNE1	MISP	PLEKHB1	1.3592	0.4761	34.1375	5.14E-09	7.71E-04
682	ITPR2	CLEC10A	SYK	1.3463	0.4874	34.1272	5.16E-09	7.74E-04
683	PTPN13	HACD4	GTF3C1	-1.4225	-0.5085	34.1262	5.17E-09	7.76E-04
684	PTPN13	CFAP53	POLA2	-1.3029	-0.4691	34.1254	5.17E-09	7.76E-04
685	POR	GNA15	MAL2	-1.3971	-0.4995	34.1241	5.17E-09	7.76E-04
686	AP1S3	SYNE1	PLEKHB1	1.4309	0.4992	34.1226	5.17E-09	7.76E-04
687	GSTP1	RECQL	UCP2	-1.3358	-0.4706	34.1113	5.20E-09	7.80E-04

688	CAPN13	CBR1	PTHLH	-1.2806	-0.4761	34.1026	5.23E-09	7.85E-04
689	CFAP74	NSG1	WNT9A	-1.3201	-0.4841	34.0988	5.24E-09	7.86E-04
690	GALNT14	RABL2A	NT5C	-1.3246	-0.4615	34.0982	5.24E-09	7.86E-04
691	ESRRG	OSBPL3	OSCP1	1.3154	0.4675	34.0844	5.28E-09	7.92E-04
692	HSPB11	CDKN1A	SERTAD1	-1.3594	-0.4865	34.0801	5.29E-09	7.94E-04
693	CENPF	APOBEC3G	LPAR5	-1.413	-0.4956	34.0772	5.30E-09	7.95E-04
694	DTX2	PEAK1	CLEC7A	-1.3103	-0.4669	34.0661	5.33E-09	8.00E-04
695	TPPP	SHANK2	WDR86	1.3185	0.4677	34.0653	5.33E-09	8.00E-04
696	RPL7A	TCF20	RPL37A	1.3648	0.5018	34.0635	5.33E-09	8.00E-04
697	MAPK10	NUCB2	CASP8	1.3317	0.4796	34.0614	5.34E-09	8.01E-04
698	DNAH5	SHANK2	ITPR3	1.2932	0.4661	34.0551	5.36E-09	8.04E-04
699	TEKT4	SHISA5	SVIP	1.3059	0.4653	34.0492	5.37E-09	8.06E-04
700	ESRRG	OSBPL3	DNALI1	1.3196	0.4774	34.045	5.39E-09	8.09E-04
701	CCDC187	PKM	SVIP	1.3456	0.4811	34.0431	5.39E-09	8.09E-04
702	CXCL6	ALOX15	LIMCH1	1.5549	0.5535	34.0425	5.39E-09	8.09E-04
703	PHLDA3	NAV2	MRTFB	-1.2892	-0.4681	34.0409	5.40E-09	8.10E-04
704	GJB3	IQCA1	KRT4	-1.4047	-0.49	34.0408	5.40E-09	8.10E-04
705	SHANK2	KCNE1	IQANK1	1.3176	0.4731	34.0397	5.40E-09	8.10E-04
706	C11orf16	SHANK2	PLXNB2	1.3348	0.4654	34.0329	5.42E-09	8.13E-04
707	STAT6	APOBEC3G	SUV39H2	-1.3217	-0.4717	34.0298	5.43E-09	8.15E-04
708	OXR1	RNF227	ADGRG6	-1.3013	-0.4757	34.0233	5.45E-09	8.18E-04
709	TNFAIP8	PRR4	NSA2	1.3409	0.4748	34.0197	5.46E-09	8.19E-04
710	MYLK	CLDN3	APBB1	-1.3542	-0.4876	34.0169	5.46E-09	8.19E-04
711	S100A9	CCDC170	SMAD3	-1.2877	-0.4642	34.0165	5.46E-09	8.19E-04
712	RUVBL1	TNFRSF19	TENT5B	-1.3156	-0.4713	34.0152	5.47E-09	8.21E-04
713	OSBPL6	A4GALT	IFT43	-1.3043	-0.4643	34.0046	5.50E-09	8.25E-04
714	GLIPR2	SLC6A15	SVIP	1.2949	0.4675	34.0045	5.50E-09	8.25E-04
715	GJB3	PIFO	PPL	-1.2988	-0.4619	34.0031	5.50E-09	8.25E-04
716	CYP26A1	GJB2	CLMN	-1.4384	-0.5145	33.9898	5.54E-09	8.31E-04
717	ESRRG	OSBPL3	C11orf49	1.3545	0.48	33.9889	5.54E-09	8.31E-04
718	SFN	REC8	POLR2A	-1.2827	-0.4682	33.9813	5.56E-09	8.34E-04
719	CYB561A3	PKM	TPRXL	-1.381	-0.474	33.9707	5.59E-09	8.39E-04
720	SPATA17	SHANK2	ITPR3	1.3407	0.482	33.9668	5.61E-09	8.42E-04
721	NUCB2	RSPH1	VNN2	1.3258	0.4758	33.9607	5.62E-09	8.43E-04
722	DNAH10	TNFRSF19	APOL1	1.2905	0.4627	33.9568	5.63E-09	8.45E-04
723	CCDC17	SHANK2	AK4	-1.347	-0.4796	33.9453	5.67E-09	8.51E-04
724	ZBBX	SHROOM3	DLK2	1.33	0.4769	33.9431	5.67E-09	8.51E-04
725	TKT	EFEMP2	GSTA1	-1.3501	-0.4786	33.943	5.68E-09	8.52E-04
726	NCALD	ALDH3B1	CEP19	1.3426	0.4804	33.9351	5.70E-09	8.55E-04
727	KIAA2012	PPP1R36	DNAJB9	-1.3404	-0.4739	33.927	5.72E-09	8.58E-04
728	PTPN6	MUC16	ADGRE5	-1.3775	-0.4892	33.9171	5.75E-09	8.63E-04

729	ESRRG	OSBPL3	RSPH4A	1.3138	0.4652	33.916	5.75E-09	8.63E-04
730	CABCOCO1	SLFN13	PLXNB2	1.3291	0.478	33.9137	5.76E-09	8.64E-04
731	ARHGEF38	ADGRF1	SYNE1	1.3963	0.5009	33.9103	5.77E-09	8.66E-04
732	GJB3	NPHP1	PPL	-1.4018	-0.5	33.9056	5.79E-09	8.69E-04
733	SHANK2	DNAJB13	AK4	-1.4239	-0.5101	33.9035	5.79E-09	8.69E-04
734	FHL2	FAM216B	POLR2A	-1.3254	-0.4692	33.8945	5.82E-09	8.73E-04
735	CEP41	C11orf16	AK4	-1.2942	-0.4663	33.8942	5.82E-09	8.73E-04
736	TCTE1	NCALD	CLIC1	1.3075	0.4692	33.8917	5.83E-09	8.75E-04
737	SHISA5	ODF3B	PLXNB2	-1.3322	-0.4731	33.8825	5.85E-09	8.78E-04
738	TEKT2	SHANK2	NAA40	1.3299	0.4792	33.878	5.87E-09	8.81E-04
739	KLHL6	TSPAN6	CCNO	1.3487	0.4822	33.8775	5.87E-09	8.81E-04
740	SFN	PNKD	EPN3	-1.3942	-0.4984	33.8761	5.87E-09	8.81E-04
741	SHROOM3	NAT1	DLK2	1.334	0.4806	33.8727	5.88E-09	8.82E-04
742	CAPN13	INKA1	MS4A8	-1.3459	-0.4759	33.8701	5.89E-09	8.84E-04
743	ATP10B	CAMK1D	PRSS8	1.3596	0.482	33.8693	5.89E-09	8.84E-04
744	FHL2	DNAAF6	AGRN	-1.2933	-0.467	33.8686	5.90E-09	8.85E-04
745	CAPN13	ADH7	ANKRD65	-1.3915	-0.4983	33.8658	5.90E-09	8.85E-04
746	S100A9	RP1	ABCC5	-1.2871	-0.4658	33.8609	5.92E-09	8.88E-04
747	TKT	CD14	CARD8	1.272	0.4684	33.8574	5.93E-09	8.90E-04
748	MAD2L2	DRC3	JAG1	-1.2733	-0.4609	33.8565	5.93E-09	8.90E-04
749	SULT1E1	CSTB	SDC1	-1.3141	-0.4651	33.854	5.94E-09	8.91E-04
750	ANXA8	PRR29	ZBTB7C	-1.3158	-0.4662	33.8454	5.97E-09	8.96E-04
751	ESRRG	OSBPL3	PPP1R32	1.3514	0.4815	33.8378	5.99E-09	8.99E-04
752	FLACC1	CRACR2A	PTHLH	1.3005	0.4724	33.8356	6.00E-09	9.00E-04
753	DNAH11	GNA15	BNIPL	-1.3218	-0.4703	33.8295	6.02E-09	9.03E-04
754	GALNT14	TMEM107	ABCC5	-1.405	-0.5002	33.8215	6.04E-09	9.06E-04
755	RAB38	PTPN6	STK17A	-1.3594	-0.4918	33.8185	6.05E-09	9.08E-04
756	S100A9	PLEKHG7	ABCC5	-1.3051	-0.4805	33.8134	6.07E-09	9.11E-04
757	RPSA	SNRNP70	RWDD2B	-1.3052	-0.48	33.8085	6.08E-09	9.12E-04
758	SESN1	UBASH3B	RPL35	-1.3074	-0.4678	33.8077	6.08E-09	9.12E-04
759	C10orf67	NUCB2	ENO2	1.3036	0.4643	33.8065	6.09E-09	9.14E-04
760	S100A9	SPAG6	ABCC5	-1.3476	-0.487	33.795	6.12E-09	9.18E-04
761	ESRRG	PLEKHG7	NOS2	1.4801	0.5221	33.7942	6.13E-09	9.20E-04
762	AQP3	GRAMD2A	CDCP1	-1.287	-0.4714	33.7936	6.13E-09	9.20E-04
763	SFN	PTGER2	LPAR5	-1.3129	-0.4712	33.7893	6.14E-09	9.21E-04
764	SFN	TTC21A	JAG2	-1.3447	-0.4828	33.7892	6.14E-09	9.21E-04
765	GJB3	C20orf96	IGSF9	-1.3234	-0.4812	33.7887	6.14E-09	9.21E-04
766	CCDC17	SHANK2	CAPN5	1.3413	0.4805	33.787	6.15E-09	9.23E-04
767	JPT1	SAMHD1	ALDH1L1	1.3663	0.4888	33.7863	6.15E-09	9.23E-04
768	ADH7	LDHD	ICA1L	1.2848	0.467	33.7848	6.16E-09	9.24E-04
769	FHL2	ENKUR	ITPR3	-1.3319	-0.4885	33.775	6.19E-09	9.29E-04

770	ESRRG	OSBPL3	ENKUR	1.386	0.4737	33.7741	6.19E-09	9.29E-04
771	SDK1	PTGES	TSPAN17	-1.3284	-0.4753	33.774	6.19E-09	9.29E-04
772	LYZ	IRF9	S100A16	1.3271	0.4762	33.7717	6.20E-09	9.30E-04
773	WLS	MUC15	TMC5	1.3304	0.4771	33.7684	6.21E-09	9.32E-04
774	DTHD1	TNFRSF19	AK4	-1.4236	-0.509	33.7598	6.24E-09	9.36E-04
775	CD14	CHST15	SLC16A3	1.3704	0.4852	33.7596	6.24E-09	9.36E-04
776	ESRRG	IQCG	CLPTM1L	-1.3017	-0.4681	33.7568	6.25E-09	9.38E-04
777	TNC	RAB37	S100A6	-1.3104	-0.4722	33.756	6.25E-09	9.38E-04
778	GALNT14	BBS12	STMN1	-1.3841	-0.4883	33.7554	6.25E-09	9.38E-04
779	BCO2	CSTB	MAL2	-1.3979	-0.5045	33.7526	6.26E-09	9.39E-04
780	GLRB	PTPRT	CDKN2D	1.3266	0.4691	33.7517	6.26E-09	9.39E-04
781	ANO1	S1PR5	ITGA6	-1.32	-0.4752	33.7419	6.29E-09	9.44E-04
782	SHROOM3	ALDH3B1	DLK2	1.3262	0.4767	33.7408	6.30E-09	9.45E-04
783	SFN	TOGARAM2	JAG2	-1.3297	-0.4765	33.738	6.31E-09	9.47E-04
784	ADGRF1	PODNL1	CD200R1	-1.3298	-0.495	33.7328	6.32E-09	9.48E-04
785	ESRRG	CAPS2	ID4	-1.4579	-0.5125	33.7295	6.33E-09	9.50E-04
786	ESRRG	EFHC2	NOSTRIN	-1.3149	-0.4722	33.7183	6.37E-09	9.56E-04
787	IL5RA	TNFRSF19	PLXNB2	1.4263	0.5019	33.7166	6.38E-09	9.57E-04
788	STING1	JPT1	LRRC4	-1.4287	-0.5123	33.7093	6.40E-09	9.60E-04
789	CEP41	SLC25A14	TMED3	-1.3462	-0.4778	33.701	6.43E-09	9.65E-04
790	EMB	BCKDHB	EGFL6	1.4019	0.4777	33.6888	6.47E-09	9.71E-04
791	KIF11	APOBEC3G	RCOR1	-1.2986	-0.464	33.682	6.49E-09	9.74E-04
792	CLIC5	CSTB	RACGAP1	-1.3321	-0.4745	33.6801	6.50E-09	9.75E-04
793	TKT	GRAMD2A	B3GNT2	-1.3457	-0.4809	33.6721	6.52E-09	9.78E-04
794	PZP	CHST9	MUC20	1.3072	0.4686	33.67	6.53E-09	9.80E-04
795	TRMT1L	ESRRG	MED25	1.3967	0.4933	33.6691	6.53E-09	9.80E-04
796	IQCG	PPP1R36	DNAJB9	-1.3266	-0.469	33.6683	6.54E-09	9.81E-04
797	SHANK2	TEX26	TLCD2	1.3109	0.4642	33.6662	6.54E-09	9.81E-04
798	GJB3	MSH3	NUAK2	-1.3237	-0.473	33.6627	6.55E-09	9.83E-04
799	SHANK2	CFAP53	SRGAP3	1.344	0.4773	33.6608	6.56E-09	9.84E-04
800	TSGA10	PTGES	HEY2	-1.3737	-0.4937	33.6605	6.56E-09	9.84E-04
801	ESRRG	ZNF473	ACTG2	-1.4339	-0.5127	33.6523	6.59E-09	9.89E-04
802	ESRRG	OSBPL3	CFAP45	1.3237	0.4648	33.6508	6.59E-09	9.89E-04
803	SFN	IL5RA	JAG2	-1.3082	-0.4696	33.6503	6.60E-09	9.90E-04
804	VWA3B	CDH3	SVIP	1.3423	0.4706	33.6477	6.61E-09	9.92E-04
805	ORAI2	ITPR2	NUCB2	-1.3971	-0.4927	33.6464	6.61E-09	9.92E-04
806	TPD52L1	AKR1C3	HNRNPA0	1.3158	0.4804	33.6354	6.65E-09	9.98E-04
807	TMEM40	JAML	SORBS2	1.3355	0.4718	33.6348	6.65E-09	9.98E-04

Results, Biologically relevant three-way interaction identification

Table S2. Gene Ontology and KEGG pathway enrichment analysis information**.**

Term	GO Levels	p-value	FDR (BH)	(-logBH)
cilium assembly	7	4.09E-25	2.36E-22	21.627088
negative regulation of intracellular signal transduction	7	4.26E-05	8.12E-04	3.090443971
negative regulation of antigen receptor-mediated signaling pathway	11	1.59E-04	2.24E-03	2.650505088
arachidonic acid metabolic process	9	2.38E-04	3.01E-03	2.521341317
negative regulation of T cell receptor signaling pathway	12	2.45E-04	3.07E-03	2.512405501
long-chain fatty acid metabolic process	8	4.32E-04	4.75E-03	2.32374017
neutrophil degranulation	9	5.06E-04	5.20E-03	2.284271017
regulated exocytosis	7	5.50E-04	5.51E-03	2.258587272
neutrophil activation involved in immune response	7	5.56E-04	5.54E-03	2.256474087
negative regulation of cellular protein metabolic process	7	6.44E-04	5.97E-03	2.223888928
leukocyte degranulation	8	6.44E-04	6.00E-03	2.221874446
unsaturated fatty acid metabolic process	8	7.04E-04	6.35E-03	2.196983958
diterpenoid metabolic process	7	8.21E-04	7.12E-03	2.147595343
antioxidant activity	7	1.13E-03	8.73E-03	2.05922899
retinoid metabolic process	8	1.48E-03	1.07E-02	1.971443427
positive regulation of apoptotic process	7	1.78E-03	1.21E-02	1.918286793
regulation of phosphatidylinositol 3-kinase signaling	9	4.09E-03	2.14E-02	1.670469717
negative regulation of interleukin-2 production	8	4.17E-03	2.17E-02	1.664287615
mitotic nuclear division	7	4.50E-03	2.29E-02	1.640776014
regulation of apoptotic signaling pathway	7	5.49E-03	2.61E-02	1.583314884
regulation of T cell receptor signaling pathway	11	5.86E-03	2.75E-02	1.560000001
endopeptidase activity	7	9.10E-03	3.67E-02	1.435489233
positive regulation of intracellular signal transduction	7	9.48E-03	3.75E-02	1.426490291
leukocyte differentiation	8	9.81E-03	3.83E-02	1.416753207
negative regulation of apoptotic signaling pathway	8	1.05E-02	3.96E-02	1.402346359
positive regulation of wound healing	7	1.05E-02	3.97E-02	1.401306799
negative regulation of apoptotic process	7	1.08E-02	4.07E-02	1.390583998
positive regulation of protein localization to plasma membrane	8	1.25E-02	4.45E-02	1.35204324
keratinocyte differentiation	7	1.27E-02	4.50E-02	1.346337829
spermatogenesis	7	1.44E-02	4.89E-02	1.310938742
regulation of interleukin-2 production	7	1.48E-02	4.95E-02	1.305646967

ID	Term	p-value	FDR (BH)	(-logBH)
KEGG:00590	Arachidonic acid metabolism	3.00E-05	6.34E-04	3.20E+00
KEGG:04020	Calcium signaling pathway	7.22E-03	3.16E-02	1.50E+00

Results, Random Forest for Selecting Genes

Fig S3. The area under the receiver operating characteristic (ROC) curve (AUC). As shown, the AUC of the classifier is 0.87.

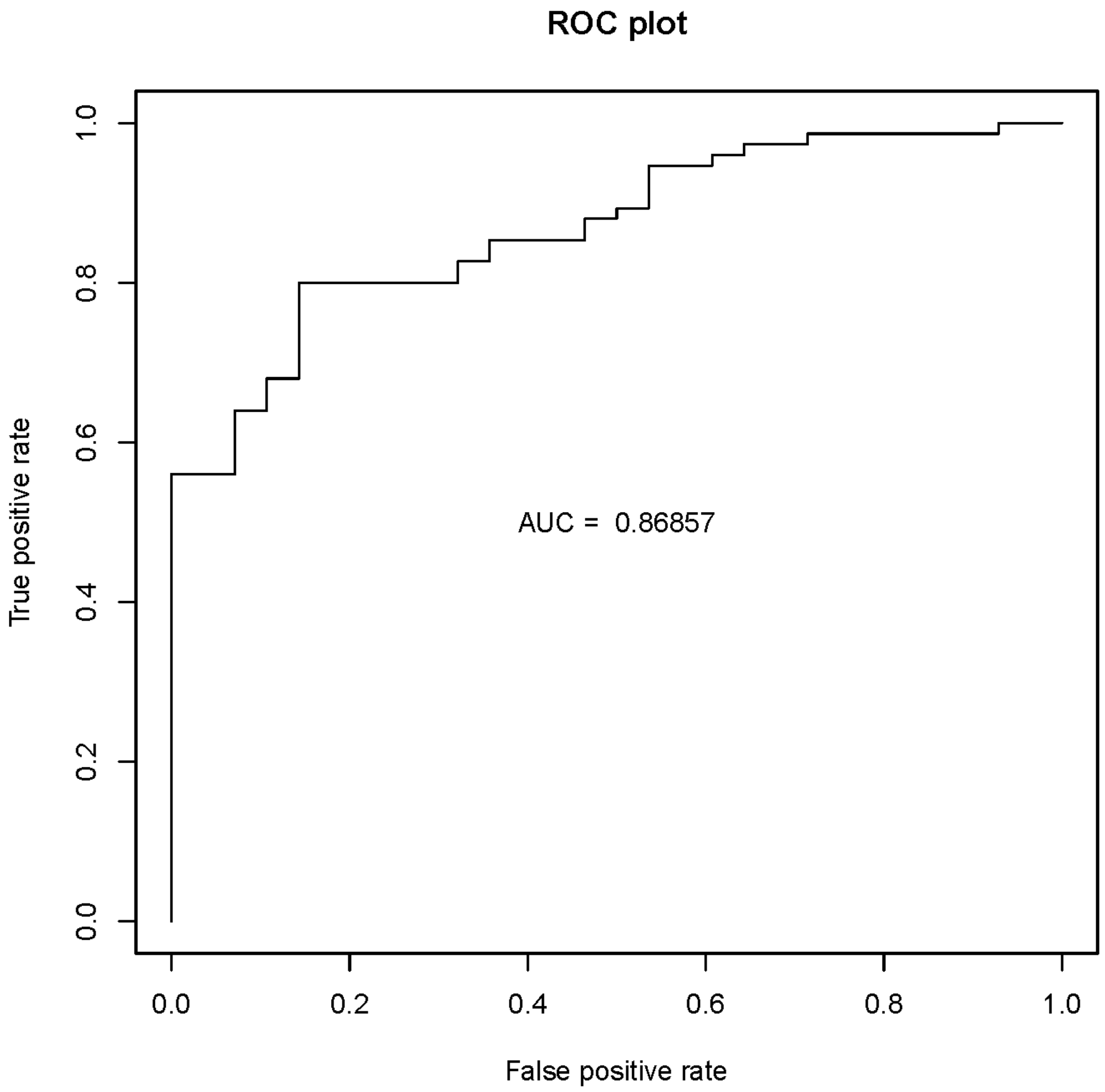

Results, Random Forest for Selecting Genes

Table S4: Analysis of Variance (ANOVA) Results for gene expression profiles of Tgfbr3 gene among three study groups: NP-NP, CS-IT, and NP-IT.

Source of Variation	Sum of Squares (SS)	Degrees of Freedom (df)	Mean Square (MS)	F (DFn, DFd)	P-Value
**Between Groups**	35.61	2	17.81	F (2, 100) = 29.87	P<0.0001
**Within Groups**	59.61	100	0.5961		
**Total**	95.22	102			

Results, Random Forest for Selecting Genes

Table S5: Analysis of Variance (ANOVA) Results for gene expression profiles of Tob1gene among three study groups: NP-NP, CS-IT, and NP-IT.

Source of Variation	Sum of Squares (SS)	Degrees of Freedom (df)	Mean Square (MS)	F (DFn, DFd)	P-Value
**Between Groups**	31.97	2	15.99	F (2, 100) = 25.27	P<0.0001
**Within Groups**	63.25	100	0.6325		
**Total**	95.22	102			

Results, Random Forest for Selecting Genes

Table S6: Analysis of Variance (ANOVA) Results for gene expression profiles of S100a9 gene among three study groups: NP-NP, CS-IT, and NP-IT.

Source of Variation	Sum of Squares (SS)	Degrees of Freedom (df)	Mean Square (MS)	F (DFn, DFd)	P-Value
**Between Groups**	24.62	2	12.31	F (2, 100) = 17.43	P<0.0001
**Within Groups**	70.61	100	0.7061		
**Total**	95.22	102			

Results, Random Forest for Selecting Genes

Table S7: Detailed gene expression profiles for three selected genes (Tgfbr3, Tob1, and S100a9).

TGFBR3	TGFBR3	TGFBR3	S100A9	S100A9	S100A9	TOB1	TOB1	TOB1
NP-NP	NP-IT	CS-IT	NP-NP	NP-IT	CS-IT	NP-NP	NP-IT	CS-IT
-0.615141	0.21864952	0.90515248	-1.76883	0.293381	-1.57444	-0.19403	1.249346	0.586273
-1.104836	0.29338123	1.42607687	0.448425	-0.70507	-1.49615	0.318639	1.574445	1.061503
-0.448425	0.16952385	1.10483574	1.303783	0.048223	-0.94208	-0.07237	0.705066	2.341027
-1.426077	-1.1503494	-0.2683089	0.52994	-0.14512	-0.42193	-0.5024	0.869424	0.768302
-1.249346	0.83477189	1.66379279	-0.19403	0.243404	-0.67449	-0.26831	1.104836	0.834772
-0.120804	-0.8010945	0.26830892	-0.61514	-0.29338	-1.0615	0.194028	1.150349	-0.14512
-1.061503	0.34410246	-0.0482231	0.318639	0.194028	-1.10484	0.475241	0.736316	0.243404
1.0615026	-0.6445316	-0.3697907	0.268309	-0.39573	-0.86942	0.145121	-0.61514	-0.80109
0.6445316	-0.2933812	-0.0723697	-0.3441	0.21865	-2.34103	-0.73632	0.096559	0.344102
-0.586273	-0.2186495	-0.5578848	-0.2434	0.705066	1.898029	0.369791	0.024105	1.898029
0.5024022	0.86942377	0.98033035	0.145121	-0.98033	-1.15035	-0.44843	-0.58627	0
-0.145121	0.14512094	-0.2434042	1.150349	-0.04822	-0.44843	-1.10484	-1.57444	0.52994
-0.736316	0.04822307	0.61514111	-0.09656	-0.21865	-0.83477	-0.52994	0.21865	0.502402
-2.069902	0.55788476	0.09655862	1.663793	-0.1208	0.169524	-1.66379	-0.70507	-0.2434
-1.768825	-0.1940281	-0.3957253	0.421929	-0.64453	-0.80109	-1.49615	-0.21865	0.615141
-0.905152	0.73631592	0.36979068	-1.02008	-1.42608	1.496147	-1.36238	1.768825	1.663793
-1.496147	0.3957253	-0.3186394	0.834772	0.736316	-0.90515	-0.98033	-0.0241	0.048223
-0.98033	-0.5024022	1.15034938	-0.5024	0.98033	0.024105	-0.67449	-1.19838	0.942076
-0.768302	0.70506589	1.02007623	-1.19838	-1.30378	-1.66379	-0.42193	-1.0615	0.644532
0.1208042	0.24340418	0.76830216	-0.7683	-0.55788	-2.0699	-0.83477	0.268309	1.303783
-1.19838	0.19402814	1.24934624	1.19838	-0.0241	0	-1.15035	-0.09656	1.19838
-1.898029	0	0.42192889	2.341027	1.574445	-1.89803	-0.64453	0.801095	0.293381
-1.362383	2.06990183	1.36238339	1.768825	-0.31864	0.07237	-1.30378	-0.16952	0.67449
-0.169524	0.80109453	1.49614688	1.061503	1.249346	-1.24935	0.448425	0.169524	2.069902
-0.475241	0.07236971	1.89802865	0.344102	-0.47524	-0.36979	-0.1208	0.905152	1.020076
-0.52994	1.76882504	1.57444497	0.502402	0.096559	-0.73632	-0.29338	-0.47524	-0.39573
-0.344102	1.1983797	0.58627265	0.67449	0.768302	-1.36238	-0.90515	0.557885	0.421929
-0.096559	1.30378267	2.34102714	0.586273	0.557885	-0.16952	-1.24935	-0.31864	0.120804
-1.020076	0.31863936		0.644532	-0.52994		-2.0699	-0.7683	
-1.303783	0.67448975		1.426077	-0.58627		-2.34103	1.426077	
0.9420758	0.02410453		0.801095	0.615141		-1.89803	1.362383	

-0.421929	-0.8347719	0.120804	0.942076	-1.76883	0.98033
-1.574445	0.44842548	0.905152	-0.07237	-0.94208	0.395725
-2.341027		2.069902		-1.02008	
-0.024105		0.869424		-1.42608	
-0.705066		1.362383		-0.86942	
0.4752408		0.395725		1.496147	
-0.67449		0.369791		0.07237	
-0.942076		1.020076		-0.36979	
-1.663793		-0.26831		-0.3441	
0.5299395		0.475241		-0.04822	
-0.869424		1.104836		-0.55788	

Reference:

1. Khayer, N., et al., *Nkx3-1 and Fech genes might be switch genes involved in pituitary non-functioning adenoma invasiveness.* Scientific Reports, 2021. **11**(1): p. 20943.
